# Replacement of feline foamy virus *bet* by feline immunodeficiency virus *vif* yields replicative virus with novel vaccine candidate potential

**DOI:** 10.1186/s12977-018-0419-0

**Published:** 2018-05-16

**Authors:** Carmen Ledesma-Feliciano, Sarah Hagen, Ryan Troyer, Xin Zheng, Esther Musselman, Dragana Slavkovic Lukic, Ann-Mareen Franke, Daniel Maeda, Jörg Zielonka, Carsten Münk, Guochao Wei, Sue VandeWoude, Martin Löchelt

**Affiliations:** 1 Department of Microbiology, Immunology, and Pathology, College of Veterinary Medicine and Biomedical Sciences, Colorado State University, Fort Collins, CO USA; 2 Department of Molecular Diagnostics of Oncogenic Infections, Research Program Infection, Inflammation and Cancer, German Cancer Research Center, (Deutsches Krebsforschungszentrum Heidelberg, DKFZ), Im Neuenheimer Feld 242, 69120 Heidelberg, Germany; 3 Clinic for Gastroenterology, Hepatology, and Infectiology, Medical Faculty, Heinrich-Heine-University Düsseldorf, Düsseldorf, Germany; 4 Present Address: Roche Glycart AG, Schlieren, 8952 Switzerland; 5 Present Address: Department of Microbiology and Immunology, Western University, London, ON Canada; 6 Present Address: University of Dar es Salaam, Dar es Salaam, Tanzania; 7 Present Address: Department of Internal Medicine II, Division of Hematology, University Hospital of Würzburg, Würzburg, Germany; 8 Present Address: Roche Pharma AG, Grenzach-Wyhlen, Germany; 9 Present Address: Division of Infectious Disease, University of Colorado, Anschutz Medical Campus, Aurora, USA

**Keywords:** Foamy virus, In vitro virus evolution, In vivo host factor requirement, Replicating vaccine vector, Lentivirus, Bet function, Vif function, APOBEC3, Restriction factor, Superinfection

## Abstract

**Background:**

Hosts are able to restrict viral replication to contain virus spread before adaptive immunity is fully initiated. Many viruses have acquired genes directly counteracting intrinsic restriction mechanisms. This phenomenon has led to a co-evolutionary signature for both the virus and host which often provides a barrier against interspecies transmission events. Through different mechanisms of action, but with similar consequences, spumaviral feline foamy virus (FFV) Bet and lentiviral feline immunodeficiency virus (FIV) Vif counteract feline APOBEC3 (feA3) restriction factors that lead to hypermutation and degradation of retroviral DNA genomes. Here we examine the capacity of *vif* to substitute for *bet* function in a chimeric FFV to assess the transferability of anti-feA3 factors to allow viral replication.

**Results:**

We show that *vif* can replace *bet* to yield replication-competent chimeric foamy viruses. An in vitro selection screen revealed that an engineered Bet-Vif fusion protein yields suboptimal protection against feA3. After multiple passages through feA3-expressing cells, however, variants with optimized replication competence emerged. In these variants, Vif was expressed independently from an N-terminal Bet moiety and was stably maintained. Experimental infection of immunocompetent domestic cats with one of the functional chimeras resulted in seroconversion against the FFV backbone and the heterologous FIV Vif protein, but virus could not be detected unambiguously by PCR. Inoculation with chimeric virus followed by wild-type FFV revealed that repeated administration of FVs allowed superinfections with enhanced antiviral antibody production and detection of low level viral genomes, indicating that chimeric virus did not induce protective immunity against wild-type FFV.

**Conclusions:**

Unrelated viral antagonists of feA3 cellular restriction factors can be exchanged in FFV, resulting in replication competence in vitro that was attenuated in vivo. Bet therefore may have additional functions other than A3 antagonism that are essential for successful in vivo replication. Immune reactivity was mounted against the heterologous Vif protein. We conclude that Vif-expressing FV vaccine vectors may be an attractive tool to prevent or modulate lentivirus infections with the potential option to induce immunity against additional lentivirus antigens.

**Electronic supplementary material:**

The online version of this article (10.1186/s12977-018-0419-0) contains supplementary material, which is available to authorized users.

## Background

Foamy viruses (FVs) are ancient retroviruses comprising the only genus of the subfamily *Spumaretrovirinae,* which are different in many aspects from the *Orthoretrovirinae* that comprise all other known retroviruses including lentiviruses (LVs) [1–3]. Despite having a wide tissue tropism in infected animals, FVs have historically been regarded as apathogenic and are endemic in primates, bovids, felids, and other hosts. Clusters of highly related viruses have been documented in closely related hosts [4–7]. While humans do not have endemic FVs, they are susceptible to zoonotic infections from non-human primates [8, 9]. FVs and LVs such as feline immunodeficiency virus (FIV) have been used to develop vectors for vaccine antigen delivery and gene therapy in a variety of mammals [10–17]. In domestic cats (*Felis catus*), feline foamy virus (FFV) and FIV establish lifelong infections despite specific host antiviral immune responses [18–21]. In contrast to FFV infection, FIV infection leads to the development of an immunosuppressive AIDS-like syndrome in some cats [18, 20, 22–24]. Thus, FVs are an attractive alternative to LV vectors due to their apathogenicity, wide tissue tropism, and establishment of a persistent infection with ongoing virus gene expression and replication [6, 7, 12, 13, 21, 25, 26]. Other advantageous features of FV-based vectors are a safer integration profile than gammaretroviral and LV vectors [11, 27], a large packaging capacity, and the ability to introduce self-inactivating properties [17, 28–31]. Investigating FV vector candidates could thus yield potential new therapies to benefit both humans and animals [16].

Both LVs and FVs are complex retroviruses encoding the canonical Gag, Pol, and Env proteins, regulatory proteins essential for replication in all cells, and accessory proteins required only in certain cells. For instance, LV Tat and FV Tas (also designated Bel1) proteins are both transactivators for virus gene expression, however, their mode of action is completely different (for review [32]). Regardless, both regulatory genes induce a positive feedback loop to generate more transactivator protein in addition to transcription of structural genes required for infectivity [32]. FVs additionally encode Bet that is generated via splicing, consisting of N-terminal Tas sequences while the majority of the protein is encoded by another reading frame, the *bel2* gene [32]. Bet is the functional homologue of the LV Vif protein, both of which are involved in countering the host intrinsic antiviral restriction factors of the APOBEC3 (A3) family [33–38].

Like all other viruses, LVs and FVs are restricted by intrinsic cell mechanisms that impair or even suppress the different phases of virus replication, progeny production, and establishment of infection in the new host (for review see [39, 40]). Nonspecific innate immunity and cell-based intrinsic immunity employing antiviral restriction factors are both absolutely required to control pathogen replication before adaptive immunity matures for long-term suppression of viral replication [41, 42]. Therefore, a fine-tuned crosstalk between innate, intrinsic, and adaptive immunity is needed to control and eliminate the pathogen as well as to build up immunological memory [41–43]. Pathogens have evolved a plethora of counteracting strategies in order to evade this control, often by the acquisition of counteracting proteins [39, 40]. The idea and concept of host-encoded restriction factors and the viral counter-defense have been in part established in human immunodeficiency virus (HIV) research. These initial studies analyzed the interplay between host-encoded A3 cytidine deaminases that result mainly in lethal mutagenesis (C to U/T exchanges) of the retroviral HIV genome during reverse transcription, and the counter-defense by LV Vif (or Bet in FVs) which result in A3 degradation (via Vif) or sequestration (via Bet) [33, 34, 36, 40, 44].

Analogous to human A3 function, feline A3 (feA3) proteins are produced in many cell types and introduce missense and stop mutations into nascent viral genomes, ultimately restricting viral replication through hypermutation and degradation [33, 34, 39]. Several studies on the function of FIV Vif and FFV Bet, which are of very different size and share no obvious sequence or structural homology [36, 38, 45], have revealed that they employ completely different modes of action to achieve the same end goal: preventing the packaging of feA3 proteins into the particle to avoid subsequent viral lethal mutagenesis. The FIV Vif protein (25 kDa) functions as an adapter molecule, binding to cognate or highly-related feA3 proteins and recruiting the ubiquitin proteasome degradation machinery, resulting in the removal of feA3 proteins from the virus-producing cell [44, 46–49]. This is the critical prerequisite to prevent cytidine deamination during or after reverse transcription of the genome. In contrast, FV Bet proteins (of 43 to 56 kDa) tightly bind A3 proteins of their cognate host species without leading to degradation, likely acting via sequestration or blocking of essential binding and multimerization sites [34, 36, 37, 45]. Therefore, *vif* and *bet* are essential viral genes required to allow productive replication in cells with active A3 expression [39, 50, 51].

Domestic cats produce multiple A3 proteins in one and two-domain forms. One-domain feA3 proteins include the A3Z2 (present as A3Z2a, A3Z2b, and A3Z2c) and A3Z3 isoforms, while read-through transcription leads to the production of two-domain feA3Z2-Z3 proteins (in A3Z2b-Z3 and A3Z2c-Z3 isoforms) [52]. These feA3 proteins have differential effects on FFV and FIV: A3Z2s markedly reduce titers of FFV lacking *bet*, while the A3Z3 and A3Z2-Z3 proteins inhibit FIV virions lacking *vif* with intermediate and high efficiency, respectively. Interestingly, both Bet and Vif counteract all feA3 regardless of whether the specific A3 isoforms efficiently restrict FFV or FIV [33, 34, 44, 46, 47, 52], suggesting a more complex relationship between these accessory genes and host restriction factor regulation than has yet been described.

Here we describe the generation and in vitro selection of FFV-Vif chimeras in which FIV *vif* partially or almost fully restored the replication capacity of *bet*-deficient FFV constructs in vitro. An in vitro-selected FFV-Vif variant that drives expression of the heterologous lentivirus Vif independent from any FFV protein and which is highly dependent on Vif expression in A3-producing cells, was used for infection of domestic cats to test the chimera’s replication competence and immunogenicity. Replication of the FFV-Vif chimera was attenuated in cats compared to wild-type FFV. Cats infected with the FFV-Vif chimera developed persistent antibody responses towards FFV proteins and FIV Vif but proviral FFV-Vif chimeric genomes were at or below the limit of detection in peripheral blood mononuclear cells (PBMC) of infected cats. In contrast, proviral genomes were consistently detected in wild-type FFV-infected cats. Inoculation of cats in the FFV-Vif chimera cohort with wild-type FFV or re-inoculation with FFV-Vif chimeric virus boosted anti-FFV Gag antibody titer following re-infection. These results suggest that compensatory changes arising in vitro seemingly allowed FIV-Vif to substitute for FFV-Bet function, but were incapable of fully supporting FFV-Vif chimeric replication competence in vivo. These findings additionally suggest the capacity of spumaviruses to superinfect cats following prior attenuated FFV replication, indicating the potential suitability of chimeric FFV as a vaccine vector in the face of a pre-existing infection and immunity.

## Results

### FIV Vif and FFV Bet confer protection from feA3 restriction in vitro

Previous studies have shown that the FIV Vif accessory protein has the capacity to direct proteasomal degradation of all known feA3 cytidine deaminase restriction factors irrespective of whether they strongly or moderately restrict FIV replication [44, 46, 47, 52]. Similarly, FFV Bet binds to all feA3 isoforms and inactivates their restriction potential by a degradation-independent, different mechanism not comparable to FIV Vif [33, 34]. In addition, FIV Vif can protect the replication capacity of *bet*-deficient FFV while FFV Bet correspondingly counteracts feA3-mediated restriction of *vif*-deficient FIV [39, 44].

To confirm here that the viral defense proteins of FFV and FIV are functionally interchangeable to protect infectivity against feA3 restriction [33, 34, 44, 46, 47, 52], transient transfection studies were conducted and representative data are shown here. First, we analyzed the susceptibility of FIVΔ*vif*-luc, a *vif*-deficient FIV luciferase (luc) expression vector (“Methods”, [44]) towards one-domain feA3Z3, and two-domain feA3Z2-Z3 isoforms (Additional file 1A). The efficacy of *luc* marker gene transduction was determined in the presence of co-transfection with FFV *bet*, FIV *vif,* or an empty control vector. Both FIV Vif and FFV Bet restored the FIV vector titer almost fully while different levels of feA3-mediated restriction were detectable only in the absence of any viral defense protein. Similarly, the replication competence of the *bet*-deleted and feA3-sensitive pCF7-BBtr FFV mutant (Table 1, [25]) was rescued by Bet and Vif. In the absence of Vif and Bet proteins, the expression of the feA3Z2b isoform strongly suppressed the titers of *bet*-deficient pCF7-BBtr (Additional file 1B). This antiviral restriction by feA3Z2b was partially or fully abrogated by co-expression of either FFV Bet or FIV Vif, respectively.

**Table 1:** Viral clones and stocks used in this study

Clones	Viral stock name^a^	Major mutation	Effect on replication (CrFK)
pCF-7 [25]	Wild-type FFV^b^	–	–
pCF7-BBtr	FFV-BBtr	Truncation at Bet amino acid 117	Fully susceptible towards feA3-mediated restriction in vitro
pCF7-BetMCS [50]	FFV-BetMCS	Insertion and replacement of Bet residues at amino acid 117 by insertion of a multiple cloning site	Fully susceptible towards feA3-mediated restriction in vitro
pCF7-Vif-4	FFV-Vif-4	Engineered Bet-Vif fusion protein	Partially susceptible towards feA3-mediated restriction in vitro
pCF7-Vif-39	FFV-Vif-39	Spontaneous frameshift	Fully susceptible towards feA3-mediated restriction in vitro
pCF7-Vif W/*1	FFV-Vif W/*1^b^	Trp to Stop mutation (TGG to TGA), unlinked *vif* gene	Enhanced, compared to pCF7-Vif-4
pCF7-Vif W/*2	FFV-Vif W/*2	Trp to Stop mutation (TGGG to TAGA), unlinked *vif* gene	Enhanced, compared to pCF7-Vif-4
pCF7-Vif W/*1 M+	–	Optimized upstream Met codon in pCF7-Vif W/*1	Similar to pCF7-Vif W/*1
pCF7-Vif W/*2 M+	–	Optimized upstream Met codon in pCF7-Vif W/*2	Similar to pCF7-Vif W/*2
pCF7-Vif W/*1 M/T	–	Upstream Met codon mutated to Thr in pCF7-Vif W/*1	Similar to pCF7-Vif W/*1
pCF7-Vif W/*2 M/T	–	Upstream Met codon mutated to Thr in pCF7-Vif W/*2	Similar to pCF7-Vif W/*2

^a^FFV-Vif variants collectively referred to as “FFV Vif chimeras”

^b^Viral stocks used in domestic cat infection experiments

### Substitution of FFV Bet by functional Vif confers FFV replication competence in feA3 expressing cells

**Fig. 1:**
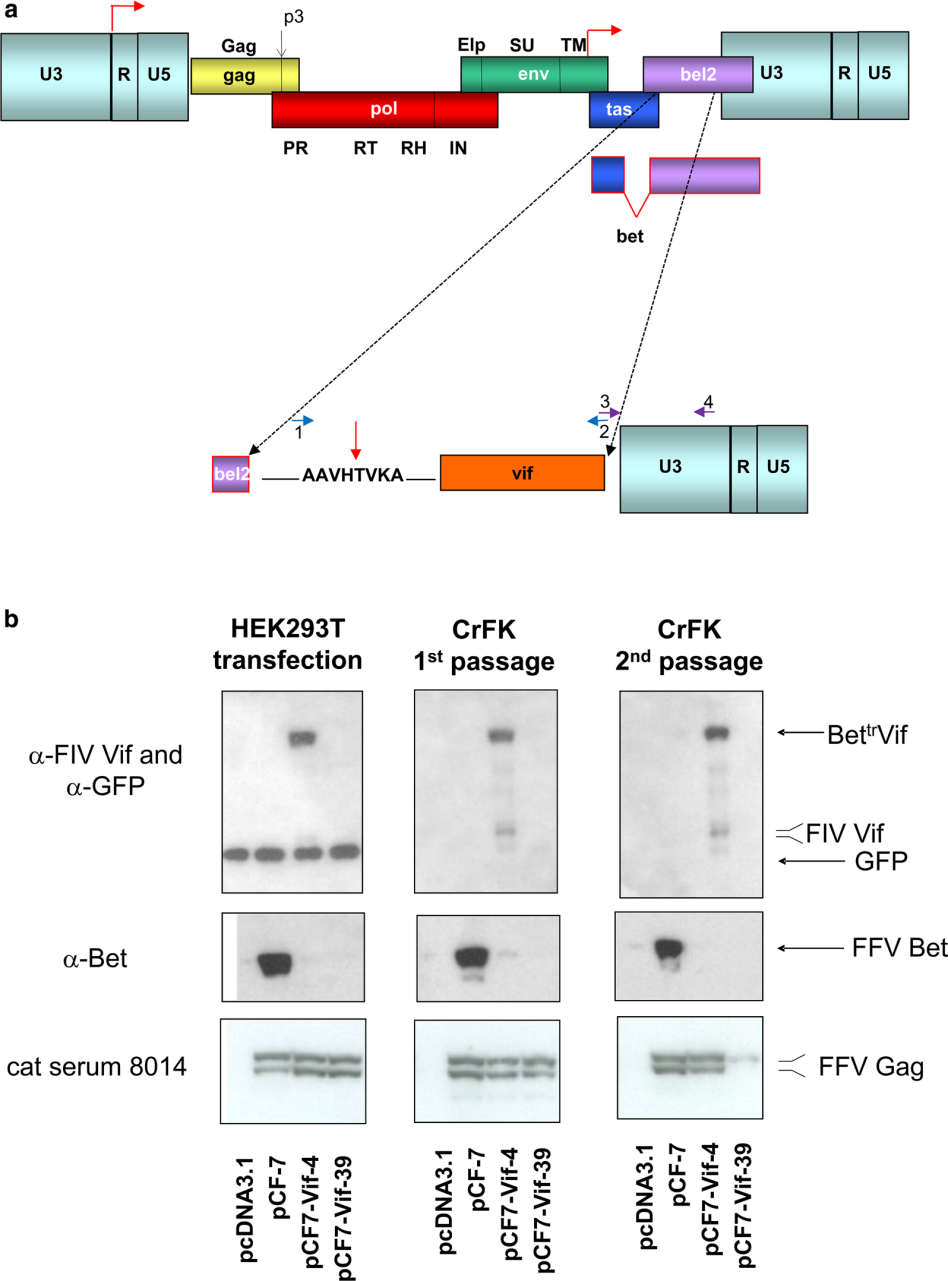
Schematic presentation of the construction of FFV-Vif chimeras and their molecular features. **a** Schematic presentation of the FFV genome with its genes and protein domains as well as the LTR and internal promoters (red bent arrows, top) and presentation of the engineered Bet^tr^Vif fusion protein (bottom). The non-functional N-terminus of *bet* (purple) was fused in-frame to the codon-optimized FIV *vif* gene including the *vif* ATG start codon. A short linker encompassing the FFV PR cleavage site (vertical red arrow, bottom) was inserted between the N-terminus of Bet and Vif. Primer pairs used to insert the *vif* gene into the FFV genome are shown in blue and violet and with numbering in the bottom panel. **b** HEK 293T cells were transfected with wild-type pCF-7, functional clone pCF7-Vif-4, non-functional clone pCF7-Vif-39, and pcDNA3.1 control DNA. Two days after transfection, cell culture supernatants and cells were harvested as described in the “Methods” section. Cleared supernatants were used for serial passaging in feA3-expressing CrFK cells and FFV titer determination (Fig. 2a). At 3 days p.i., infected CrFK cells and supernatants were harvested and used as above. Cell lysates from transfected HEK 293T cells and CrFK cells after the first and second passage were subjected to immunoblotting against FIV Vif and co-transfected GFP, FFV Bet, and FFV Gag (cat serum 8014). The positions and names of the detected proteins are given at the right margin

To initially assess whether FFV Bet could be functionally replaced by FIV Vif, resulting in feA3-resistant FFV variants, *bet* sequences downstream of the essential *tas* transactivator gene (at Bet amino acid 117) in the full-length FFV clone pCF7-BetMCS (Table 1) [25, 50] were replaced by a codon-optimized FIV *vif* gene [44, 52] shown schematically and in detail in Fig. 1a and Additional file 2. Similar to other Bet fusion proteins engineered in the FFV proviral context [12, 25], an FFV protease (PR) cleavage site was introduced between the truncated N-terminus of Bet and the intact FIV *vif* gene start codon. Gene swapping did not affect FFV *tas*, and we have previously demonstrated that the N-terminal Bet sequence retained in the pCF7-Vif clones does not counteract feA3-mediated restriction of FFV replication [45]. Sequencing of resultant clones was conducted to confirm the genetic identity and correctness of the newly created clone pCF7-Vif-4 (Table 1). A spontaneous frame shift mutation arose in subclone pCF7-Vif-39 (Table 1), abrogating Bet^tr^Vif fusion protein expression completely, making this clone suitable for use as a negative control.

**Fig. 2:**
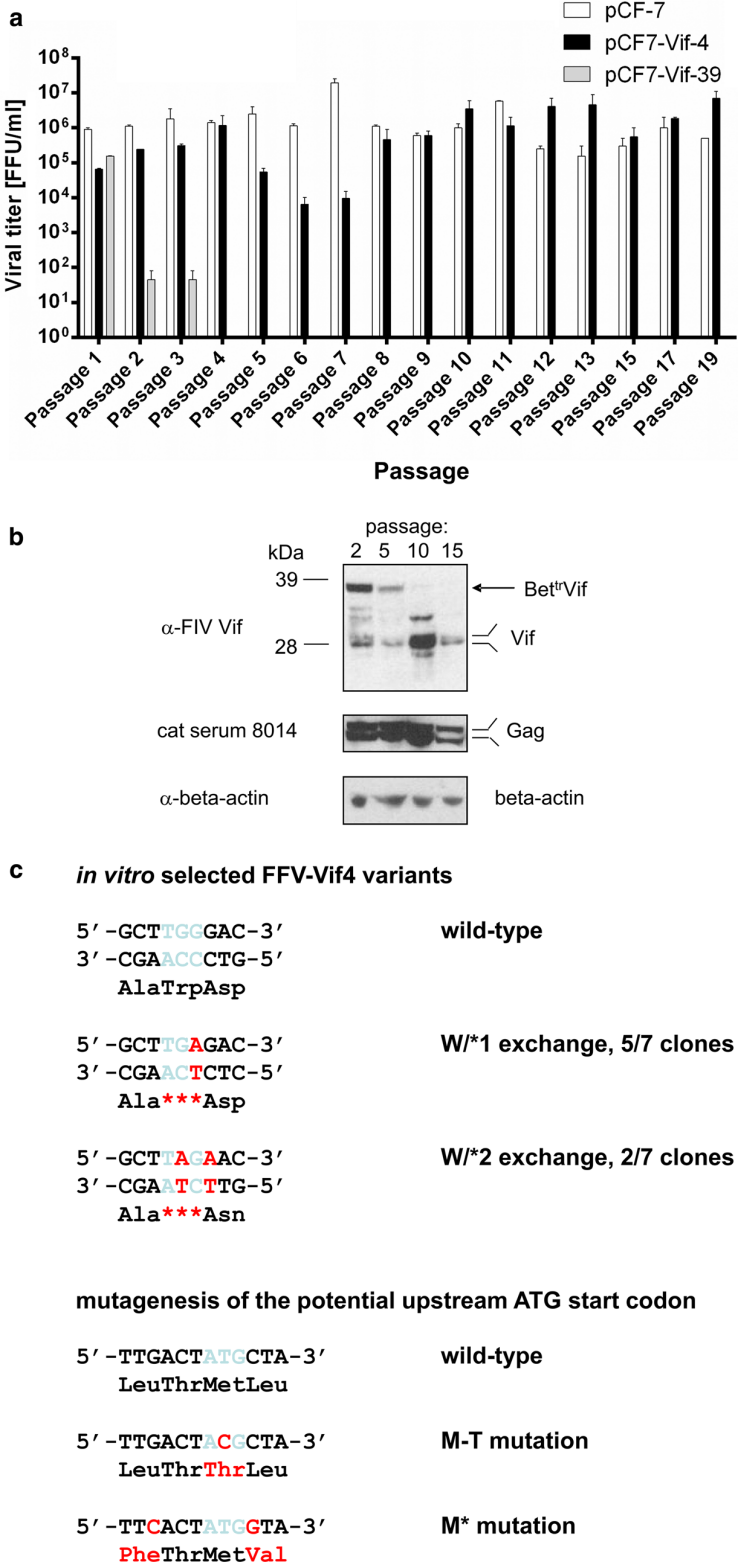
In vitro selection and molecular characterization of pCF7-Vif-4 variants with increased replication competence. Plasmids pCF7-Vif-4, -39, and pCF-7 were transfected into HEK 293T cells. Two days after transfection, cell-free supernatants were inoculated on CrFK cells and serially passaged twice a week on CrFK cells (every 3 or 4 days) as described above for Fig. 1b. **a** FFV titers were determined in duplicate using FeFAB reporter cells and are shown as bar diagram for selected passages over time. Error bars represent the standard deviation. **b** Selected cell extracts from the CrFK passages were subjected to immunoblotting. The immune-detection with a Vif-specific antiserum initially showed mainly the engineered Bet^tr^Vif and the proteolytically released Vif, then various unidentified Vif variants, and finally (passages 10 and 15) predominantly the authentic Vif protein. FFV Gag proteins were detected in all samples as expected using cat antiserum 8014 while in the bottom panel the β-actin loading control is shown. **c** Sequence context of the in vitro-selected W/* mutations (light blue original Trp to the stop codon in red) suggests feA3 editing of the minus strand of FF7-Vif-4-derived reverse transcription intermediates in the PyPyC sequence context (top panel, Py = pyrimidine residue). Below, mutagenesis of the in-frame ATG 14 codons upstream of the *vif* gene is shown only for the sense strand (bottom panel). The ATG start codon is shown in light blue and the engineered residues and changes amino acids are in red

Plasmids pCF7-Vif-4, pCF7-Vif-39, and parental wild-type FFV full-length pCF-7 genome (Table 1) were transfected into human embryonic kidney (HEK) 293T cells. Supernatants were passaged twice on Crandell feline kidney (CrFK) cells (known to express feA3 [52]) to assess the ability of the chimeras to replicate in feline-origin cells. The full-length Bet^tr^Vif fusion protein and the mature Vif processing products were stably expressed by clone pCF7-Vif-4 which was, as expected, not the case for the frame shift mutant pCF7-Vif-39 (Fig. 1b, top panel). FFV Bet was only expressed by the wild-type pCF-7 genome upon transfection and serial passages (Fig. 1b, middle panel). Similar amounts of full-length FFV p52^Gag^ and the processed p48^Gag^ were synthesized by pCF7-Vif-4 and wild-type pCF-7 in transfected HEK 293T and infected CrFK cells while in clone pCF7-Vif-39, Gag expression was almost lost at the second CrFK cell passage (Fig. 1b, bottom panel). The loss of Gag expression of clone pCF7-Vif-39 was paralleled by a very rapid decline of infectivity (Fig. 2a). In contrast, titers of pCF-7 were higher than those of pCF7-Vif-4 and none of them showed a sharp decline of viral infectivity. These data indicate that intact FIV *vif*-chimeric pCF7-Vif-4 is replication-competent in feA3-positive CrFK cells, albeit at lower efficiency than wild-type FFV (Fig. 2a).

### Passage through CrFK enhances FFV-Vif chimera replication efficiency

We continued passaging progeny of wild-type pCF-7 and chimeric pCF7-Vif-4 (see above, Fig. 2a) for 20 passages in order to use in vitro selection and evolution to obtain FFV-Vif variants with higher replication capacity in the presence of the feA3 proteins endogenously expressed in CrFK cells [34]. During the first seven passages, wild-type pCF-7 displayed titers between 10^6^ and 10^7^ focus-forming units per ml (FFU/ml) (Fig. 2a). During this phase, infectivity of the chimeric clone pCF7-Vif-4 was approximately one to two logs lower (10^4^–10^6^ FFU/ml). Starting at passage eight, however, titers of pCF7-Vif-4 progeny approached that of wild-type pCF-7, indicating emergence of pCF7-Vif variants with enhanced replicative ability in vitro (Fig. 2a). Selected samples harvested during CrFK passaging were analyzed for FFV Gag and Vif expression (Fig. 2b). FFV Gag expression was consistently detectable in all cell lysates using FFV reference serum from cat 8014 (Fig. 2b, middle panel). Early, during viral passages 2 and 5, the Bet^tr^Vif fusion protein and its proteolytic cleavage products were the primary Vif-reactive proteins detectable. At passage 10, Bet^tr^Vif became undetectable and the Vif protein of approximately 25 kDa was detected, along with additional Vif-reactive bands of higher molecular mass. At passage 15, mostly Vif proteins in the 25 kDa size range were identified (Fig. 2b, top panel).

To detect potential adaptive genetic changes in the FFV genome, DNA was prepared from FFV-Vif-4-infected CrFK cells at passage 18 and used as template for PCR to amplify and clone the complete *bet*^*tr*^*vif* region. In seven of nine amplicons, a tryptophan codon (TGG, Trp) located in the *bet* sequence 50 codons upstream of the *vif* ORF had mutated to become TAG and TGA stop codons (Fig. 2c and Additional file 2). These changes were transitions of either the first or second G residue to an A (Fig. 2c, top panel) yielding two different stop codons, indicated by an asterisk (*), as either a TGA (five out of seven sequences, designated W/*1) or a TAG stop codon (two out of seven sequences, designated W/*2). In the five clones that had incorporated the TGA stop codon in the *bet* sequence, a G172R mutation in the overlapping Tas-coding sequence occurred. In the two TGG to TAG mutants, a G residue following the TGG codon was also changed to A (i.e. TGGG was altered to TAGA). This resulted in a G172L exchange in Tas and a D/N change directly downstream of the new *bet* stop codon. All nucleotide exchanges correspond to C/T exchanges of the antisense strand in a sequence context PyPyC (Fig. 2c; Py = pyrimidine residue), corresponding to the canonical A3 mutation context in retroviral genomes [34, 53]. Additional genetic changes were not consistently detected in the *bet*^*tr*^*vif* region.

### Unlinking *vif* from *bet* by Trp/stop mutagenesis is essential for increased infectivity

The importance of the identified Trp/stop (W/*) mutations upstream of the *vif* sequence was analyzed using reverse genetics. Both W/* mutations in the *bel2* linker sequence upstream of *vif* were inserted into the original pCF7-Vif-4 to determine whether they represent adaptive mutations increasing the titer of the corresponding FFV-Vif chimera. These clones were named pCF7-Vif W/*1 (TGG/TGA) and pCF7-Vif W/*2 (TGG/TAG, Table 1).

An additional outcome of the W/* mutations was the “emergence” of an in-frame ATG codon between the new W/* stop codon and the authentic *vif* start codon (Additional file 2). To test whether this ATG codon could serve as an alternative translational initiation codon for the inserted *vif* gene, this Met ATG was replaced in the engineered pCF7-Vif W/*1 and -W/*2 clones and the parental pCF7-Vif-4 clone by a threonine (Thr) codon (suffix M/T, see Fig. 2c lower panel and Table 1). In addition, and as a complementing strategy, the surrounding nucleotide sequence of this ATG codon was converted to an optimal Kozak translational initiation context sequence (GCCA/GCCATGG, start codon underlined, [54]) as shown in Fig. 2c, lower panels. The corresponding clones are labeled by the suffix M + (Table 1). The M/T mutation resulted in a silent mutation at the *tas* C-terminus while the change to a Kozak sequence resulted in two amino acid exchanges in *tas* at the C-terminus, i.e. D206H and A208G, and, in addition, a leucine to phenylalanine (L/F) exchange upstream, and a leucine to valine (L/V) exchange directly downstream of the potential Met start codon in the linker sequence (see Fig. 2c).

Transient co-transfection studies using a luc FFV LTR reporter construct together with either a CMV-IE promoter-driven Tas expression clone and a CMV-IE-driven β-gal plasmid or the FFV genomes pCF-7, pCF7-Vif-4, pCF7-Vif W/*1, and the different M/T and M + derivatives thereof were conducted. While the CMV-IE promoter-driven Tas expression clone yielded very high luc activities, the genomic wild-type and chimeric proviral FFV clones described above did not show significant differences in Tas transactivation, indicating that the mutations introduced do not significantly influence overall transactivation and gene expression (Additional file 3).

Clones pCF7-Vif W/*1 and -W/*2 and the different M/T and M + derivatives were transfected into HEK 293T cells and supernatants were tested for the replication competence of the FFV-Vif chimera in feA3-positive CrFK cells by serial CrFK cell passaging as described above. Serial passaging after either 60 or 84 h (Additional file 4A and 4B) showed similar outcomes: the pCF-7-encoded wild-type FFV had slightly higher titers (about fivefold) than mutants pCF7-Vif W/*1 and -W/*2 and their derivatives. For these clones and the corresponding M/T and M + clones, titers were stable during serial passages. This was not the case for the original pCF7-Vif-4 clone encoding the Bet^tr^Vif fusion protein, where titers steadily and reproducibly declined upon serial passages in several independent experiments. The data show that both W/* mutations in the FFV *bet* sequence upstream of the *vif* gene cause in feA3-expressing CrFK cells a clear increase of replication competence compared to the pCF-Vif-4 encoding the Bet^tr^Vif fusion protein. However, the replication competence of the pCF7-Vif W/*1 and -W/*2 clones was slightly lower than that of the wild-type FFV genome pCF-7. In addition, the FFV-encoded, in-frame ATG codon located 14 codons upstream of *vif* is probably not used as a start codon for Vif protein expression since its replacement by a Thr codon, or the optimization of the surrounding residues towards more efficient translational initiation, did not significantly affect viral titers.

### Reduced steady state levels of feA3Z2b by FFV-Vif chimeric clones pCF-Vif-4 and pCF7-Vif W/*1 and -W/*2

**Fig. 3:**
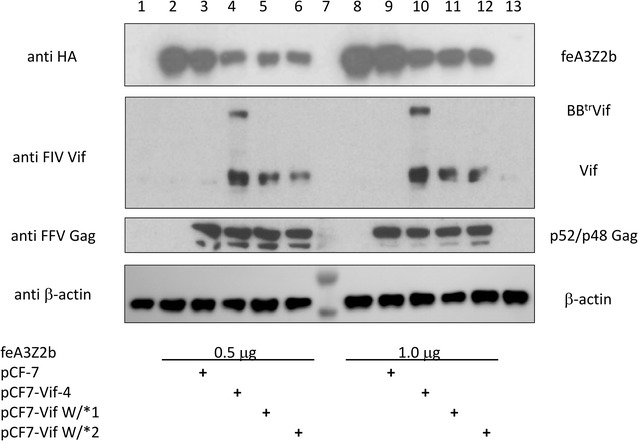
Reduced steady state levels of feA3Z2b in FIV Vif- and Bet^tr^Vif-expressing cells. Parental wild-type FFV full-length pCF-7 genome and FFV-Vif chimeric clones pCF-Vif-4, pCF7-Vif W/*1, and -W/*2 were transfected into HEK 293T cells together with 0.5 (Fig. 3, lanes 2 to 6) or 1.0 μg (lanes 8 to 12) of a plasmid encoding HA-tagged feA3Z2b as indicated below the blots. Cells transfected with the plasmid encoding feA3Z2b and pcDNA, as well as pcDNA-transfected cells served as controls (lanes 2 and 8, and 1 and 13, respectively). Cells were lysed 2 d after transfection and 20 μg total of each protein lysate was subjected to immunoblotting against HA (detecting HA-tagged feA3Z2b), FIV Vif, FFV Gag and β-actin (from top to bottom and indicated at the left). Lane 7 was loaded with a pre-stained protein marker. The bands corresponding to apparent molecular masses of 40 and about 55 kDa are seen below and above the β-actin of 42 kDa (bottom panel developed in an Intas ECL Chemocam Imaging device). All other blots were exposed to autoradiography films and thus, pre-stained protein markers are not visible in lane 7. The names of proteins specifically detected by immunoblotting are given at the right-hand side

Co-transfection experiments were conducted to study whether the steady state levels of feA3Z2b are decreased by Bet^tr^Vif fusion protein or the authentic Vif encoded by FFV-Vif chimeric clones pCF-Vif-4 or pCF7-Vif W/*1 and -W/*2, respectively (Table 1). As indicated in Fig. 3 (bottom panel), parental wild-type FFV full-length pCF-7 genome and FFV-Vif chimeric clones pCF-Vif-4, pCF7-Vif W/*1, and -W/*2 were transfected into HEK 293T cells together with a plasmid encoding HA-tagged feA3Z2b (the major feA3 restriction factor of Bet-deficient FFV) [33, 34]. Cells transfected with the plasmid encoding feA3Z2b and pcDNA as well as pcDNA-only-transfected HEK 293T cells served as controls. Cellular antigens were harvested two d after transfection and subjected to immunoblotting (Fig. 3). The control blots conducted confirm proper loading of samples (anti β-actin, bottom panel) and comparable expression of FFV proteins in wild-type and chimeric FFV provirus-transfected samples and Bet^tr^Vif fusion proteins and FIV Vif by FFV-Vif chimeric clones pCF-Vif-4 and pCF7-Vif W/*1 and -W/*2, respively (anti FFV Gag and anti FIV Vif, middle panels). As expected and previously shown [33, 34], the steady-state levels of HA-tagged feA3Z2b were not significantly affected by co-expression of wild-type FFV expressing Bet (anti HA, top panel, compare lanes 2 to 3 and 8 to 9). In stark contrast, levels of HA-tagged feA3Z2b were reproducibly and strongly reduced in cells expressing either Bet^tr^Vif and/or authentic FIV Vif (compare lane 2 to 4, 5, and 6 and lane 8 to 10, 11, and 12 in Fig. 3, top panel). In another and independent experiment with a highly similar outcome, only co-transfection of CMV-IE promoter-based and codon-optimized FIV Vif expression plasmids reduced feA3Z2b to undetectable levels (data not shown). In summary, the data clearly support the conclusion that the Vif protein in the FFV-Vif chimeric clones leads to decreased steady state levels of feA3Z2b, most probably via proteasomal degradation [33, 44, 46, 47, 52].

### Experimental infection of cats with chimeric virus FFV-Vif W/*1

**Fig. 4:**
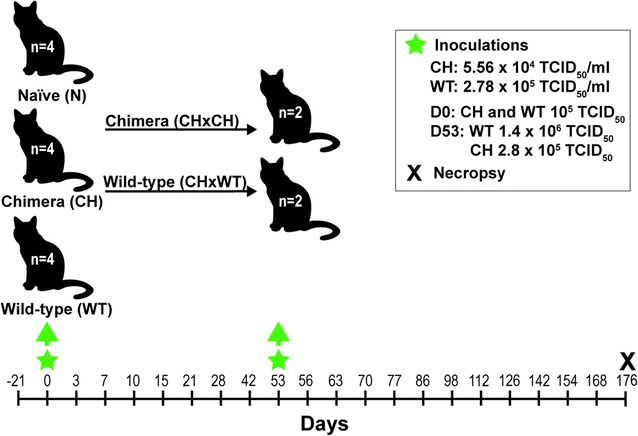
Experimental infection of cats with wild-type FFV and the FFV-Vif W/*1 chimera. Twelve SPF cats were separated into groups (n = 4 each) based on the inoculum type administered at day 0: naïve (N), wild-type FFV (WT), and chimeric FFV-Vif W/*1 (CH). Cats received 10^5^ TCID_50_ of either wild-type or chimera. Animals were monitored daily for clinical signs of infection and blood samples were collected on days specified on the timeline above to characterize infection and immune responses. Samples were collected for baseline data on day -21. On day 53, cats in the CH group were re-inoculated with either undiluted wild-type FFV of 2.78 × 10^5^ TCID_50_/ml (n = 2, referred to as CH1WT and CH2WT) or undiluted FFV-Vif W/*1 of 5.56 × 10^4^ TCID_50_/ml (n = 2, referred to as CH3CH and CH4CH). Inoculation time points are marked by green stars. Animals were humanely euthanized and necropsied on day 176 p.i. (black X)

To investigate whether the FFV-Vif chimera with the Bet-independent expression of Vif is replication-competent and immunogenic in cats, we performed inoculation experiments with FFV-Vif W/*1 (Table 1). This clone was selected for in vivo infection studies since it is the major variant detected in our in vitro experiments and is caused by only a single nucleotide exchange from the original engineered pCF7-Vif-4 chimera. Cats were separated into naïve (N), wild-type (WT), or chimeric (CH) groups based on inoculum type. The timeline of inoculations, sample collections, and final necropsy are shown in Fig. 4. None of the cats displayed signs of clinical illness or hematologic changes indicative of disease throughout the duration of the study.

### Wild-type inoculated cats exhibited persistent FFV DNA proviral loads in PBMC in contrast to chimera-inoculated cats

**Fig. 5:**
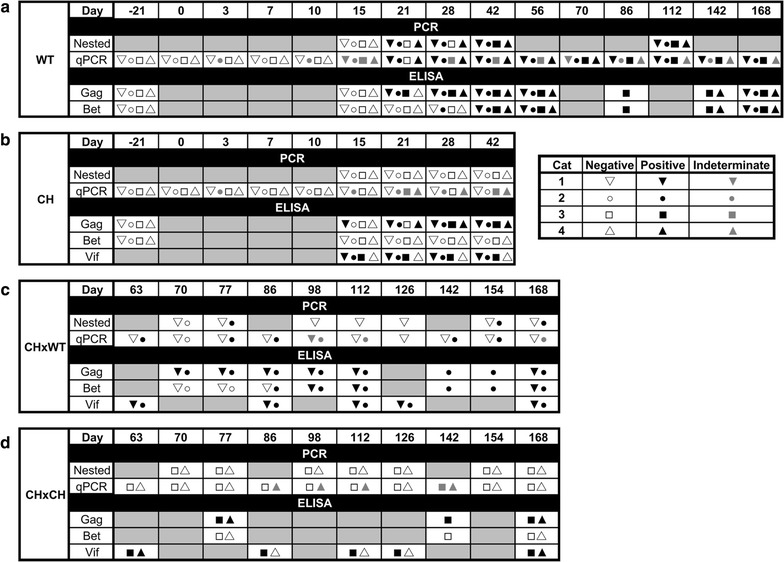
Results of PCR and ELISA assays over the entire study period. Summary of real time quantitative and nested PCR (qPCR and nPCR, respectively) on PBMCs and ELISAs for FFV Gag and Bet and FIV Vif performed before and following inoculation as given in the panels. The same symbols were used for cats 1–4 in WT and CH groups. Only the cats for which symbols are present (see inserted legend) were tested at the corresponding time point. Gray boxes represent time points where animals were not tested. CH-group cats were re-inoculated on day 53 (not shown) as described in “Methods”. **a** WT group results (days -21 to 168 p.i.). **b** CH group results (days -21 to 42 p.i.). **c** CH×WT group results (days 63 to 168 p.i.). **d** CH×CH group results (days 63 to 168 p.i.)

**Fig. 6:**
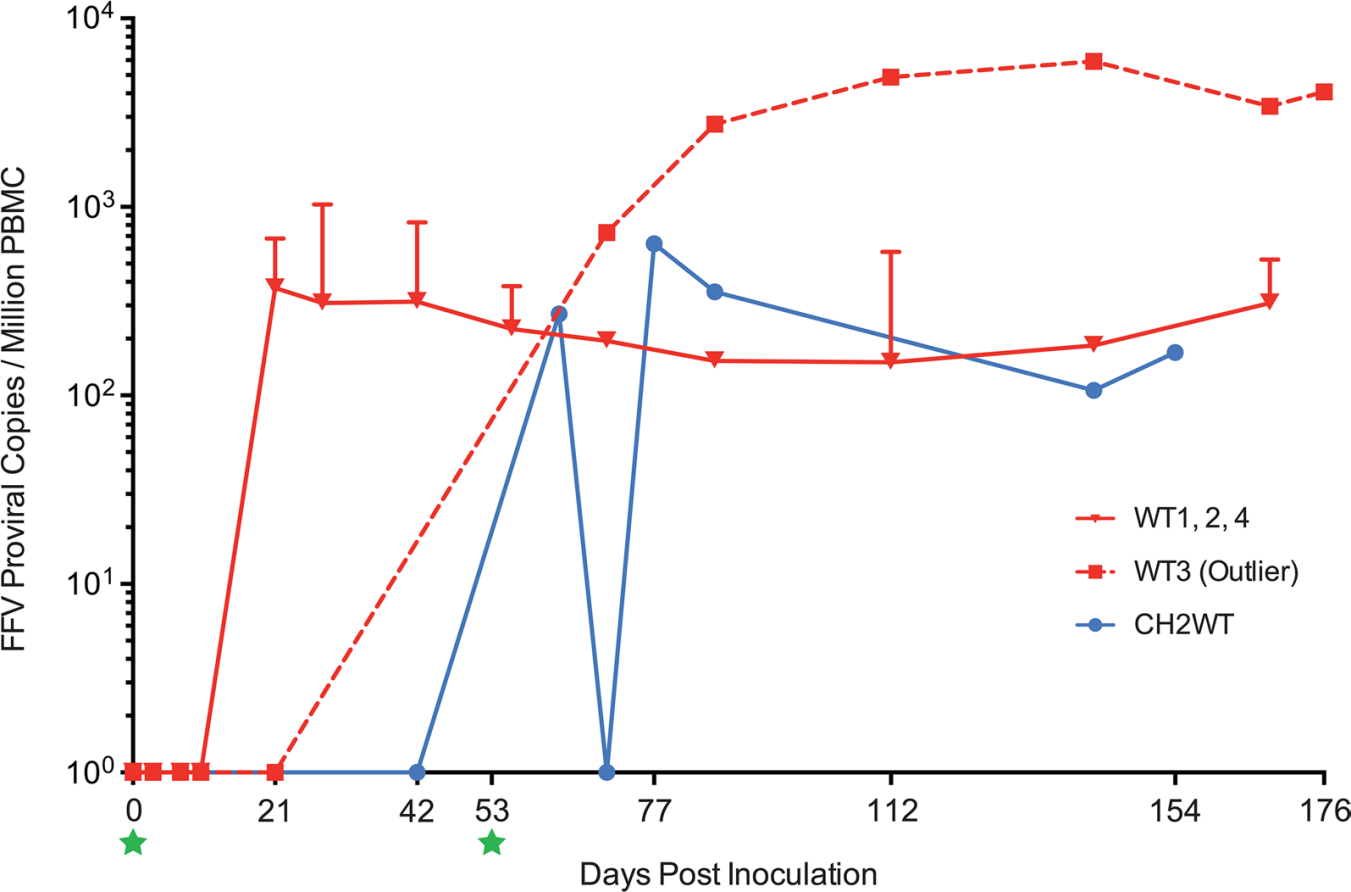
Wild-type FFV inoculated cats developed persistent infection of PBMCs. Real time quantitative PCRs (qPCR) were performed on PBMCs following inoculation on day 0 (left green star). The solid red line illustrates the proviral load mean of three WT-inoculated cats with similar viral kinetics. These cats had detectable PBMC FFV DNA on day 21 p.i. by both qPCR and nested PCR and developed persistent proviral loads between 100 and nearly 1500 copies per million PBMC. The dotted red line displays a different PBMC FFV DNA pattern observed in cat WT3 (“outlier”) which was not PCR-positive until 42 days p.i. (nPCR, see Fig. 5a). This individual had a mean proviral load 1–2 logs higher than the other WT cats and almost 6000 viral copies per million PMBC at peak viremia. The blue line represents cat CH2WT, which was re-inoculated with wild-type virus on day 53 p.i. (right green star). This was the only re-inoculated cat to test unambiguously positive on day 63 p.i. (qPCR). The other cat in this cohort (CH1WT) and the two cats in the CH×CH group are not represented in the graph due to indeterminate qPCR and negative nPCR results (see “Methods” and Fig. 5c and d). Naïve cats were completely PCR-negative throughout the study and are also absent on this graph. Error bars represent standard deviation

To compare viral load and kinetics between inoculation groups, we evaluated the presence of FFV proviral DNA in PBMC over time (Figs. 4, 5). Naïve control cats remained absolutely PCR-negative at all time points tested (Additional file 5). Cats in the WT group developed a persistent PBMC proviral load as early as 21 days post-infection (p.i.) (Figs. 5a, 6), while indeterminate PCR reactions were detected earlier (Fig. 5a and Additional file 5). By day 42 p.i., all WT cats were PCR positive and positivity was consistently detected throughout the rest of the study (Fig. 5a). Cat WT3 (subsequently also referred to as “outlier”) had a PBMC FFV DNA pattern that differed from the rest of the WT cohort (Fig. 6). This animal was not PCR-positive until day 42 (vs. day 21 as in its cohort-mates). Throughout the rest of the study, the outlier cat’s overall viral load was however much higher (highest at 5920 viral copies/10^6^ cells on day 142 p.i.) than the other WT cats (WT2 had the highest viral load at 1230 viral copies/10^6^ cells on day 28 p.i.) (Fig. 6).

Three out of four cats inoculated with only FFV-Vif chimeric virus (CH group) showed indeterminate results for FFV PBMC provirus DNA by qPCR analysis at some of the time points tested prior to re-inoculation on day 53 p.i. (Fig. 5b and Additional file 5). One of the chimera-inoculated cats re-inoculated on day 53 with wild-type virus (cat CH2WT) demonstrated FFV proviral DNA in PBMC 24 d post re-inoculation, while the other cat in this cohort remained indeterminate or negative throughout the study (Fig. 5c). The highest viral load recorded for cat CH2WT was 656 viral copies/10^6^ cells 24 days post re-inoculation (Fig. 6). Both cats re-exposed to the FFV-Vif chimera displayed repeated indeterminate PCR results in blood before and after superinfection (Fig. 5b, d).

### Gag-specific immune reactivity in infected animals confirms replication competence of wild-type FFV and FFV-Vif chimera

**Fig. 7:**
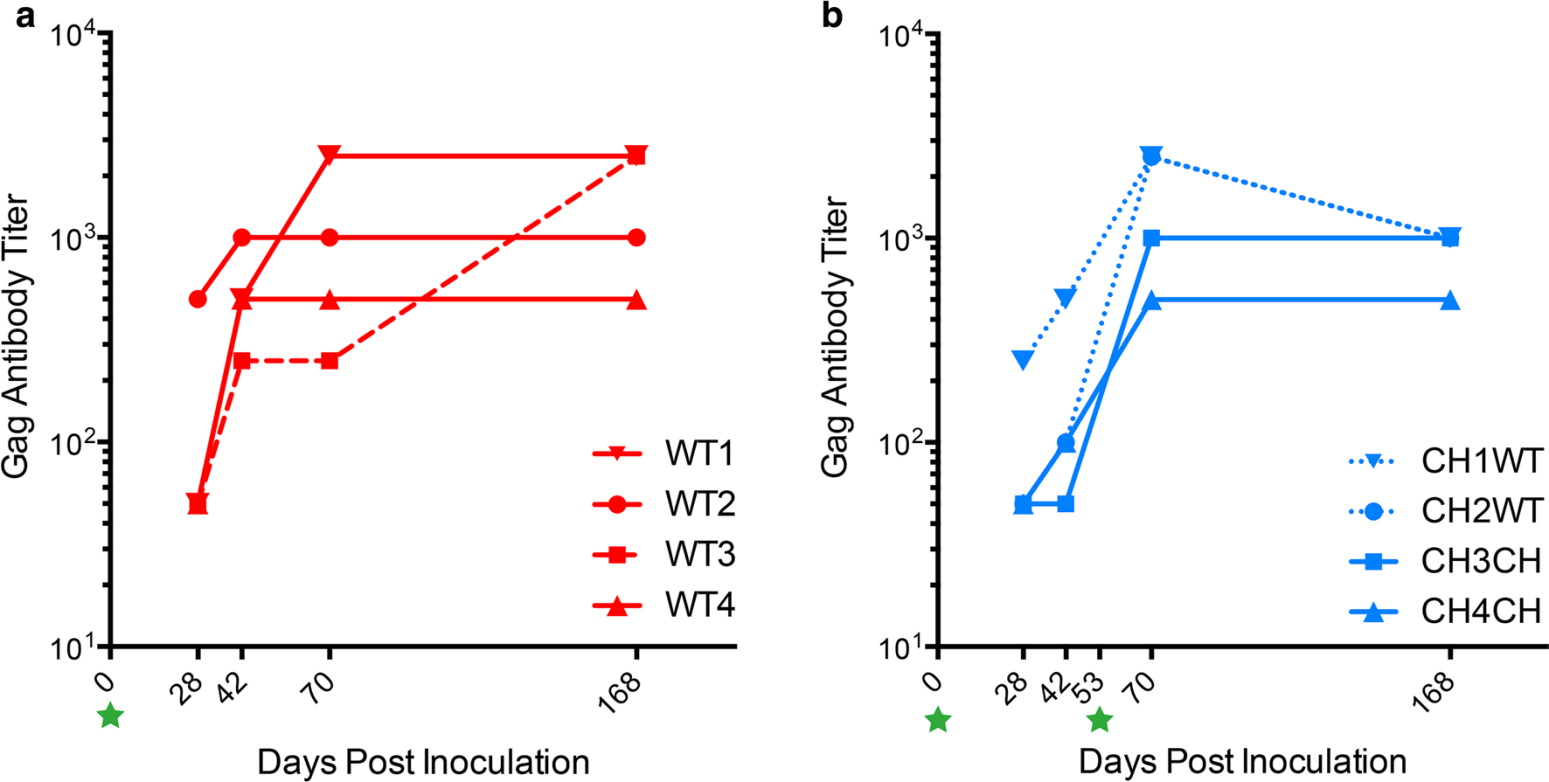
Cats infected with wild-type FFV and FFV-Vif W/*1 developed FFV Gag-specific immunoreactivity. A GST-capture ELISA was performed to evaluate antibody response to FFV infection. **a** Anti-Gag antibody titers in WT cats on days 28, 42, 70, and 168 p.i. The dotted red line represents WT3, the outlier cat. Animals displayed rising levels of antibody by day 42 which either continued to increase over time or plateau. **b** Anti-Gag antibody titers in CH cats that were re-inoculated with wild-type (CH×WT, dotted lines) or FFV-Vif W/*1 chimera (CH×CH, solid lines). These cats similarly had increasing anti-Gag antibodies around day 42 that continued to increase or plateau following re-inoculation. In order to detect low-level reactivity, sera were assayed at a 1:50 dilution leading to some reactivities which were out of the linear range of the assay

All FFV-infected cats strongly seroconverted against Gag while all naïve control animals were negative (Additional file 6, reactivity at 1:50 dilution). In order to determine the kinetics and strength of anti-Gag reactivity, selected serum samples from wild-type FFV and FFV-Vif-infected animals were analyzed before and after superinfection (only cats in the CH group received a second inoculation, Fig. 7a, b). Wild-type FFV-infected cats had detectable specific anti-Gag antibody responses as early as 21 or 28 days p.i. (Fig. 5a and Additional file 5). Antibody levels for these cats continued to increase to final titers between 500 and 2500 (Fig. 7a). FFV Gag antibodies of FFV-Vif-infected animals were first detected by day 15 p.i. (Fig. 5b and Additional file 5) and increased gradually until superinfection, after which Gag-specific titers were attained that were equivalent to wild-type-infected cats (Fig. 7a, b). Anti-Gag reactivity was detected in all four CH group cats at approximately the same seroconversion rate as wild-type FFV-infected cats, though titers tended to be lower prior to re-exposure in the CH group (Fig. 7b and Additional file 5).

### Infected cats seroconverted against accessory FFV Bet and FIV Vif proteins

**Fig. 8:**
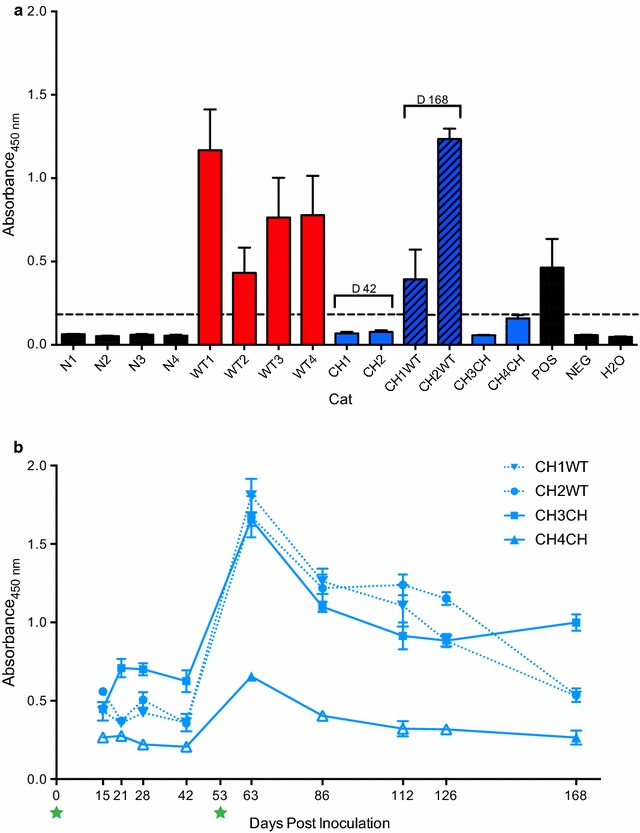
Animals inoculated with wild-type FFV or FFV-Vif chimera seroconverted to FFV Bet or FIV Vif. Antibody response against FFV Bet and FIV Vif antigens were measured by antibody capture ELISAs as described in the “Methods” section. **a** Anti-Bet antigen reactivity for each animal at final time points unless specified. WT cats (red bars), and cats that received chimera and then wild-type FFV (CH×WT, black and blue striped bars) seroconverted against Bet. Animals exposed to only FFV-Vif W/*1 (cats CH1 and CH2 prior to day 53, and CH3CH and CH4CH, solid blue bars) were negative for anti-Bet antibodies as expected. Black bars show naïve cats, and positive and negative control samples. **b** Three out of 4 animals inoculated with chimeric virus developed a detectable anti-Vif immune response as early as 15 days p.i. Antibody response increased following re-inoculation for all animals, causing a detectable response in the fourth animal (CH4CH), though sero-reactivity was low compared to other animals for this individual, and only rose above positive cutoff absorbance on days 63 and 168. Filled shapes indicate positive ELISA absorbance values compared to negative controls (> 2 standard deviation above the mean of duplicate negative samples), whereas open triangles for CH4CH indicate ELISA absorbance values below positive cutoff. Values reported represent mean of duplicate samples and bars indicate standard deviation

All cats infected with wild-type FFV only (WT 1–4) or FFV-Vif plus wild-type FFV (CH1WT and CH2WT) demonstrated substantial FFV Bet sero-reactivity by day 168 p.i. (Figs. 5a, c, 8a, and Additional file 5). As observed in previous studies [12, 21], Bet-specific antibodies appeared slightly later than Gag sero-reactivity (Fig. 5 and Additional file 5). Naïve controls and cats CH3CH and CH4CH were Bet-antibody negative as expected. Most importantly, Vif reactivity in three out of four FFV-Vif-infected animals was clearly positive at day 42 p.i., prior to superinfection on day 53 p.i. (Fig. 8b). Surprisingly, Vif-specific reactivity in these animals was detectable by day 15 p.i. despite the fact that qPCR did not detect provirus (Fig. 5b and Additional file 5). Re-inoculation of these cats with either wild-type FFV (animals CH1WT, CH2WT) or FFV-Vif chimera (CH3CH) resulted in a boost in Vif sero-reactivity at day 63 p.i. Animal CH4CH, which showed no Vif reactivity prior to superinfection, exhibited only transient FIV Vif reactivity after re-exposure (Fig. 8b).

## Discussion

This study describes the generation of replication-competent variants of FFV that express FIV Vif in lieu of FFV Bet. An engineered FFV genome expressing a fusion protein of a non-functional N-terminal Bet domain fused to the full-length Vif was clearly attenuated in vitro. Second-generation FFV-Vif chimeras expressing the authentic codon-optimized *vif* gene showed much higher *vif*-dependent replication competence in feA3-expressing cells, only slightly decreased in vitro compared to wild-type FFV. In experimentally infected cats, replication of the chimeric FFV-Vif variant was attenuated but led to the induction of FFV Gag-specific antibodies together with those directed against the engineered heterologous FIV Vif protein. Importantly, cats infected with the FFV-Vif chimera could be superinfected with wild-type FFV or the chimera, in both cases resulting in a strong immunological boost of sero-reactivity against FFV and FIV Vif.

The successful replacement of FFV *bet* by FIV *vif* in the context of the FFV genome may have been aided by two mechanisms. First, a codon-optimized and thus Rev-independent FIV *vif* gene was inserted, allowing for efficient translation of the Vif protein [32]. Second, LV Vif proteins function as catalytic regulators of proteasomal feA3 degradation; therefore, much lower amounts of fully functional Vif may be required to inactivate A3 activity compared to FV Bet, which acts stoichiometrically via direct binding to the feA3 protein [33]. Thus, the attenuated replication of the initially constructed pCF7-Vif-4 chimera was likely due to high expression levels of the functionally impaired Bet^tr^Vif fusion protein.

In support of the hypothesis that Bet and Vif are differentially expressed in vivo, Bet sero-reactivity is high and has diagnostic value in infected cats and bovines [55, 56]. In contrast, while anti-Vif antibodies have been described in HIV patients [57, 58], Vif has not been shown to be a major humoral immune target of FIV infection, and seroconversion against Vif has not been well studied in FIV infection (personal communication, Dr. Chris Grant).

Apparently, inhibitory effects of either the complete N-terminal part of Bet plus the linker sequence, or the N-terminal residues of the linker residues present downstream of the engineered FFV PR cleavage site (see Fig. 1a) favored the emergence of Trp/stop (W/*) variants. This is strongly suggested by the fact that two independent, yet highly related mutational events led to the W/* mutation in the linker sequence upstream of *vif.* The reverse genetic experiments conducted do not support translational initiation at the upstream Met residue located in the linker sequence as important for Vif protein expression. We thus assume that in the in vitro-selected clones, fully functional Vif is expressed from its authentic start codon, though the exact mechanism by which FIV Vif protein is expressed from pCF7-Vif W/*1 and pCF7-Vif W/*2 is unknown. We assume that internal re-initiation of protein biosynthesis may be involved, but other mechanisms cannot be excluded. While the mechanism of Vif expression of clones pCF7-Vif W/*1 and pCF7-Vif W/*2 is unknown, all FIV Vif proteins engineered into the FFV genome including the first-generation clone pCF7-Vif-4 encoding the Bet^tr^Vif fusion protein lead to dramatically reduced steady state levels of feA3 proteins as shown for the major restriction factor of FFV, feA3Z2b (see Fig. 3).

In line with the assumption that the original Bet^tr^Vif fusion protein conferred suboptimal protection against feA3 restriction, both mutations leading to the adaptive W/* mutations occurred in a sequence context of the negative strand that is indicative of feA3 editing, suggesting the chimeric viruses did not confer robust protection against feA3. Both mutated C residues of the negative strand are preceded by C residues in the sequence 5’-TC**CC**-3’ (deaminated C residues in bold face letters, see also Fig. 2c), and therefore should function as optimal feA3 substrates, however, alternative mutational pathways might have also played a role. The fact that suboptimal feA3 inhibition leads to adaptive changes induced by feA3 DNA deamination supports our proposed concept that the heterologous and functionally relevant transgene FIV *vif* is essential for efficient propagation of the replicating virus, and thus confers a strong selective advantage by protecting against feA3 restriction. Consequently, the transgene *vif* has to be stably maintained in the absence of *bet* during serial passages, as demonstrated in Fig. 2b. The importance of the *vif* transgene for FFV-Vif replication is further underscored by the fact that additional adaptive changes, such as unlinking from N-terminal Bet sequences, were required to restore full biological activity as an inhibitor of feA3 restriction.

The advantage of adapting this replication-competent FV vector system as a vaccine delivery vehicle is that the immunogen Vif is essential for replication, and should be thus stably maintained by the engineered vector. Further, LV Vif has been shown to elicit T and B cell reactivity in HIV-infected individuals [59–63]. A corresponding PFV-based replicating vector system carrying the HIV *vif* gene may therefore be an interesting vector for the development of anti-HIV immunotherapies.

The in vivo wild-type FFV inoculations confirm that experimental infection of outbred, immunocompetent cats with clone pCF-7-derived wild-type FFV leads to a persistent infection with consistent detection of FFV proviral DNA in PBMC and a strong sero-reactivity against Gag and Bet proteins, similar to other reports [12, 21]. In contrast, animals inoculated with FFV-Vif W/*1 remained either proviral DNA negative or indeterminate throughout the study based on nested and qPCR analysis of PBMC DNA (Fig. 5b, d). Surprisingly, despite the inability to unambiguously detect FFV provirus in cats exposed to FFV-Vif W/*1, clear sero-reactivity against FFV Gag and heterologous Vif protein were detected after primary inoculation (Figs. 5, 7). This observation is consistent with previous studies in different FVs that serology is much more sensitive for the identification of exposed animals than PCR-based studies using PBMC [21, 55, 64, 65].

FFV in vivo infection experiments were conducted with wild-type FFV or chimeric FFV-Vif W/*1. This resulted in detectable, but low, proviral load in wild-type-infected animals and either undetectable or indeterminate proviral loads in cats infected with the FFV-Vif chimera. It is feasible that the exchange of Bet for Vif altered tissue tropism and site of viral replication in FFV-Vif W/*1 exposed cats, and this contributed to the inability of tracking viral infection via peripheral blood PCR. While initially either negative or indeterminate based on PCR results, cat CH2WT was superinfected with wild-type FFV on day 53 p.i. and showed a productive PBMC FFV infection on a similar timeline after inoculation as the wild-type-infected animals.

The animals in the chimeric cohort did not seroconvert against Bet as anticipated but they displayed clear anti-Vif antibody responses starting at day 15 p.i., demonstrating that substituting Bet by Vif elicited specific immune responses. Given that anti-Vif antibodies have not been widely reported during FIV infection, our findings may indicate replication is occurring in cells or cell compartments where it is more easily recognized as a foreign antigen. Antibody production against Vif was initially not robust, but following re-inoculation with both wild-type and FFV-Vif chimeric virus, anti-Vif antibody response markedly increased (Fig. 8b). Following re-inoculation of the FFV-Vif-infected animals with wild-type FFV virus, both cats initially infected with FFV-Vif W/*1 also produced anti-Bet antibodies, demonstrating that infection with the chimera did not protect against subsequent infection with wild-type FFV (Figs. 5c, 8a).

The data document that, despite a lack of consistent detection of FFV provirus DNA, FFV-Vif W/*1 is able to induce persistent antibody responses in domestic cats that were boosted by re-inoculation. As noted above, we were able to document superinfection with two highly related FFV variants. Clinical evaluations following exposure to wild-type FFV or chimera suggests that FFV produces an apathogenic infection during the study period. Further studies should be conducted to understand chronic FFV infection and potential associated pathologic changes as well as the possibility of worsened pathology following superinfection with other FFV serotypes or other viral infections [66]. It would also be important to challenge wild-type FFV-infected cats with chimeric virus to determine whether infection with wild-type FFV may induce neutralizing immunity thus preventing superinfection with an attenuated FFV vaccine construct.

It cannot be ruled out that the antibody response detected in FFV chimera-infected animals was related to exposure to viral inoculum versus actively replicating virus, since PCR results were indeterminate. However, anti-Vif antibody production in three out of four FFV-Vif chimera-inoculated cats detected throughout the monitoring period, and an anti-Gag response equivalent to wild-type antibody titers is supportive of the conclusion that low-level viral replication occurred [21, 55, 64, 65]. Whether or not FFV-Vif W/*1 replicated poorly or not at all, the fact that pCF7-Vif W/*1 was highly replication-competent in CrFK cells but strongly attenuated in vivo suggests that Bet may play a currently unknown critical role in viral replication competence in vivo in addition to antagonizing A3-mediated restriction. Here, inactivation of other components of the host’s innate or intrinsic immunity as well as an essential co-factorial role for the replication in specific cell types in vivo are plausible reasons for the attenuated phenotype. Alternatively, other aspects of the manipulated pCF7-Vif W/*1 genome may impede replication in the native host. Further studies may elucidate additional complex host-virus restriction pathways that are relevant in vivo but are functionally masked or not relevant during in vitro infections.

Findings presented here illustrate a role for pCF7-Vif W/*1 to be used as a novel anti-LV vaccine delivery scaffold. This system would exploit a non-pathogenic vector that has to stably retain the Vif vaccine antigen and may be a therapeutic option to boost immunity towards an existing HIV infection in order to eliminate infected cells. The option to insert additional B and T cell epitopes at the terminus of the truncated Bet may be a means to extend and direct the host immune response towards additional epitopes (Slavkovic Lukic and Löchelt, unpublished observations). The ability to administer repeatedly or simultaneously the FV-based vaccine vector, directing expression of additional or newly acquired antigens, is an additional strength of our system as low level or absence of replication would hinder use of pCF7-Vif W/*1 as a vector delivery system that requires greater viral replication. Our results suggest that prior infection with wild-type FFV might not impair response to FFV-Vif, though superinfection studies will need to be conducted before this vector could be commercially developed. Experiments determining the viability of FV-LV Vif chimeric variants would also have to include assays to determine stability and functionality of inserted heterologous epitopes. Since we have documented that seroconversion occurs against Vif and Gag during FFV-Vif W/*1 exposure in the absence of intentional adjuvation, the attenuated replication does not impair its use as an antigen expression platform for eliciting antibodies against foreign antigens, and could even improve its biological safety.

## Conclusions

Our in vitro and in vivo studies show the feasibility of constructing a replicative FFV-Vif vector that incorporates FIV Vif and replaces FFV Bet protein expression to counteract intrinsic feline A3 restriction factors. The FFV-Vif chimera inoculation of domestic cats induced a specific immune response against the heterologous Vif protein which under superinfection boosted antibody production against both FFV Gag and FIV Vif. Superinfection was also possible using wild-type FFV as evidenced by seroconversion against FFV Bet in animals initially inoculated with the chimeric construct, which provides plausibility of using this vector in domestic cat populations which may already be infected with wild-type virus. These findings demonstrate that this and additional FV vector systems may be further studied to develop potential therapeutic or preventive avenues against lentiviral infections including HIV.

## Methods

### Cells, culture conditions, and DNA transfection

Crandell feline kidney (CrFK) cells were used for FFV infection and propagation [34, 66, 67]. Human embryonic kidney (HEK) 293T cells used for plasmid transfection were propagated as described [68]. FeFAB cells (CrFK-derived cells that carry a ß-galactosidase gene under the control of the FFV LTR promoter that is activated via FFV infection and subsequent Tas expression) were used to determine viral titer as described previously [68]. PBMC were purified from feline blood using Histopaque gradients (Sigma Aldrich, St. Louis, MO). HEK 293T cells were transfected or co-transfected by using a modified calcium phosphate method described previously [68]. In serial passage experiments, wild-type pCF-7 and Vif-chimera pCF-Vif-4 were transfected into HEK 293T cells [13]. Supernatants were harvested 2 days post transfection and used to infect feA3-positive CrFK cells. Supernatants from these infections were serially passaged twice a week (every third or fourth day p.i.) to new, uninfected CrFK cells. A total of 20 serial passages were conducted.

### FFV propagation and titration

For viral propagation of wild-type FFV and chimera (generated by transfection of HEK 293T cells), 10^6^ CrFK cells/ml were seeded and infected at a multiplicity of infection (MOI) of 0.1. Supernatants were harvested and used for viral titer estimation and further viral propagation. FFV titers were determined using 5 × 10^4^ FeFAB cells/well grown in 24-well plates and infected with serial 1:5 dilutions as described [13]. Titers were calculated by determining the highest dilution that contained blue-colored infected cells through light microscopy.

### Wild-type and FFV-Vif chimera viral propagation and titration for cat infections

2 µg of FFV pCF-7 [25] or pCF7-Vif W/*1 plasmid were transfected into CrFK cells using Lipofectamine and supernatants were harvested for amplification in CrFK cells. Microscopic observation of cells was conducted daily and considered to be infected if they displayed cytopathic effects (CPE) of vacuolization, cytomegaly, and multinucleation [69–71]. Supernatants of infected cells were harvested and frozen on 2, 6, 9, and 13 days p.i. CPE end-point dilution titration was conducted on CrFK cells to determine TCID_50_/ml. CrFK (3 × 10^4^ cells/well) were incubated with 25 µl of virus-containing supernatants in five-fold dilutions from the aforementioned days and observed for CPE up to 17 days p.i. The number of CPE-positive wells was used to determine TCID_50_/ml using the method of Reed and Muench [72]. Supernatants that yielded the highest titers were selected for animal inoculations.

### FIV titration system and FFV LTR luc reporter assay

Production of FIV luc reporter viruses, normalization according to reverse transcriptase activity, and target cell infection and reporter readout were done as previously described [44]. FFV reporter assays using co-transfection of HEK 293T cells with the full-length FFV LTR luc reporter plasmid pFeFV-LTR-luc and the different FFV chimeras generated in this study or the FFV Tas expression construct pFeFV-Bel1 were conducted as described previously [73].

### Molecular cloning

Replacement of FFV *bet* coding sequences by a codon-optimized FIV *vif* gene in the FFV provirus vector pCF7-BetMCS, which carries a multiple cloning site directly downstream of *bet* without affecting *tas* [50], was done via fusion PCR cloning using the proof-reading *Pfu* polymerase as specified by the supplier (New England Biolabs, Frankfurt Germany) [13]. For PCR primer sequences, see Table 2. In brief, the codon-optimized *vif* gene was first amplified using a sense primer with upstream sequences encompassing a terminal *Nhe*I site, followed by a *Sac*II site and the sequence encoding the FFV protease (PR) cleavage sequence AAVHTVKA (see Fig. 1a, and Additional file 2) directly fused in-frame to the start codon of *vif* while the antisense primer was complementary to the terminal *vif* sequence followed by an *Age*I restriction site (Fig. 1a, bottom panel, pair of blue primers, # 1 and 2). The other amplicon was generated with a sense primer also containing an *Age*I site and annealed to FFV sequences about 120 nt upstream of the essential FFV poly-purine tract while the antisense primer was downstream of a unique *Sph*I site in the U3 region of the FFV LTR (Fig. 1a, bottom panel, pair of violet primers, # 3 and 4). The amplicons generated were fused in a third PCR using only the sense primer of the first and the antisense primer of the second reaction (primers # 1 and 4). The amplicon was digested with *Nhe*I and *Sph*I and inserted into pCF7-BetMCS [50] digested with *Nhe*I and *Sph*I. The resulting clone pCF7-Vif was analyzed by DNA restriction analysis and DNA sequencing. Similarly, site-directed W/* mutagenesis in pCF7-Vif-4 and mutagenesis of the methionine codon and its flanking sequences in pCF7-Vif W/*1 and -W/*2, were done using PCR primers shown in Table 2. The resulting fragments were inserted into the clones pCF7-Vif W/*1 and pCF7-Vif W/*2 via three component ligations using unique *Bsp*EI, *Nhe*I and *Xho*I restriction sites.

**Table 2:** Primers used for cloning and PCR detection

Primer name	Sequence (5′–3′)
*pCF7*-*Vif cloning*	
FFV-Vif #1	GCGGGCTAGCGCCGCGGTACACACCGTCAAAGCCATGAGCGAGGGGACTGGCAG
FFV-Vif #2	GTGCTCTCCAAAGACCGGTTATCACAGCTCGCCGCTCCACAGCAGATTCC
FFV-Vif #3	GGCGAGCTGTGATAACCGGTCTTTGGAGAGCACAAGCTGATG
FFV-Vif #4	CGCTCTGTTGCATGCCG
*Mutagenesis of the upstream start codon*	
FFV 9233-F	GCGGTCCGGAACACCCAAGACGGATCCTACTCG
M/T-R	CGGC*GCTAGC*TCTAGTTAG*CGT*AGTCAAATCCCTCTCCCCAC
M+-R	CGGC*GCTAGC*TCTAGTTAC*CAT*AGTGAAATCCCTCTCCCCAC
*PCR amplification of in vitro*-*selected FFV*-*Vif variants*	
FFV 9366-F	CCACTTCTGTTTGGACCTTACC
FFV-10288-R	CAGCTTGTGCTCTCCAAAGC
*Nested FFV PCR*	
FFVgag-F1	CTACAGCCGCTATTGAAGGAG
FFVgag-R1	CCCTGCTGTTGAGGATTACC
FFVgag-F2	TTACAGATGGAAACTGGTCCTTAGT
FFVgag-R2	CATCAGAGTGTTGCTGTTGTTG
*Real*-*time quantitative PCR*	
FFVgag -F	GGACGATCTCAACAAGGTCAACTAAA
FFVgag-R	TCCACGAGGAGGTTGCGA
FFVgag-TM	AGACCCCCTAGACAACAACAGCAACACT

### Cloning and sequencing of in vitro selected FFV-Vif variants

DNA from CrFK cells infected with in vitro selected variants of pCF7-Vif-4 was harvested at passage 18 using the DNeasy extraction kit as specified by the supplier (Qiagen, Hilden, Germany). Sense primer FFV 9366 and antisense primer 10288 (Table 2) were used to amplify a 923 nt fragment of the *bel1*–*vif* region. Amplicons were cloned into pCR-TOPO TA vectors (Invitrogen, Karlsruhe, Germany) and subjected to in-house Sanger DNA sequencing of both strands.

### Animals and experimental design

Twelve specific-pathogen-free (SPF) cats, aged 6–8 months and negative for common feline pathogens including FFV and FIV, were obtained from the Colorado State University (CSU) SPF Colony and housed in an Association for Assessment and Accreditation of Laboratory Animal Care International-accredited animal facility at CSU. All procedures were approved by the CSU Institutional Animal Care and Use Committee prior to initiation of the study. Cats were separated into three groups (n = 4 per group) based on inoculation type: FFV-negative CrFK culture media (naïve, N), wild-type FFV (WT), or chimeric FFV-Vif W/*1 (CH) (Fig. 4). Virus-inoculated animals received 10^5^ TCID_50_ in 2 ml under ketamine anesthesia, split into 1 ml intramuscularly (i.m.) and 1 ml intravenously (i.v.). Cats were monitored daily for clinical signs of disease, and body temperature and weight were measured weekly. Peripheral blood was collected via cephalic or jugular venipuncture and processed to obtain serum and PBMC. On day 53 p.i., all cats in the CH cohort were re-inoculated each with 5 ml of undiluted virus (wild-type virus 2.78 × 10^5^ TCID_50_/ml or chimeric virus 5.56 × 10^4^ TCID_50_/ml, split into 1 ml i.m., 2 ml i.v., and 2 ml subcutaneously). Two of these cats were re-inoculated with wild-type FFV virus (henceforth referred to as CH1WT and CH2WT) and the other two cats with FFV-Vif W/*1 (now referred to as CH3CH and CH4CH). Animals were humanely euthanized for necropsy on day 176 p.i. (Fig. 4).

### Nested and real-time quantitative PCR assays

Nested FFV PCR (nPCR) was performed on PBMC DNA to screen for initial infection status. Proviral DNA was purified and amplified using 0.5 µM *gag*-specific forward and reverse primers listed in Table 2 under the following cycling conditions for the first round of nPCR: 94 °C for 2 min, 35 cycles of 94 °C for 30 s, 57 °C for 30 s, 72 °C for 1 min, and a final elongation step at 72 °C for 5 min. For the second round, 2 µl of first-round product was added to the reaction and amplified in these conditions: 94 °C for 2 min, 35 cycles of 94 °C for 30 s, 57 °C for 30 s, 72 °C for 30 s, and 72 °C for 5 min. Products were electrophoresed in 1.5% agarose gel in Tris-acetate buffer and stained with GelRed™ Nucleic Acid Gel Stain (Biotium, Hayward, CA) then visualized to look for the 333 base-pair PCR product. Real-time quantitative PCR (qPCR) was performed in triplicate on viral DNA as previously described [64] using 0.5 uM forward and reverse *Gag*-based primers and 0.1 uM probe (Table 2) with the following modified conditions: 95 °C for 3 min, 45 cycles of 95 °C for 10 s, and 60 °C for 40 s. Viral copy number quantification was based on a plasmid standard curve prepared from plasmid pCF-7. FFV-Gag real time PCR assay sensitivity is 1–10 viral copies per reaction [64]. Infection status was divided into 3 categories: positive, negative, and indeterminate. Animals considered unequivocally “positive” had qPCR results with Cq values less than or equal to 37 in 2–3 out of the three reactions, consistent with viral load greater than 10 copies/reaction. Animals considered “negative” were negative for all triplicate tests (this included all naïve cats and “no template” controls at all defined times). Animals classified as “indeterminate” had qPCR replicates with Cq values > 37, equivalent to 0–10 copies per well. Indeterminate copy number calculations were not used in Fig. 6 since values obtained were below the assay’s lower limit of quantitation.

### Gag, Bet and Vif immunoblotting

Cell lysate from FFV-infected CrFK cells or transfected HEK 293T cells were subjected to immunoblot analyses as described [13, 21]. Identical amounts of proteins were separated by SDS-PAGE, blotted, and reacted against different anti-FFV sera. FFV Gag and Bet proteins were detected by rabbit anti-Gag polyclonal serum (1:3000 dilution) and rabbit anti-Bet polyclonal serum (1:2500 dilution) [13]. FIV Vif was detected by a mouse anti-FIV-Vif antibody (NIH AIDS repository, Maryland, USA) at a 1:500 dilution. Membranes were incubated with secondary anti-rabbit polyclonal antibodies or anti-mouse IgG (Sigma, Munich, Germany) conjugated to horseradish peroxidase (1:5000 to 1:2000 dilution) and visualized by chemiluminescence (ECL Western Blot Kit, Amersham Buchler, Braunschweig, Germany). Blots were then probed against actin using mouse anti-actin antibody (1:8000 dilution, Sigma).

### GST-capture ELISA for detection of Gag and Bet seroconversion

GST-capture ELISA was performed to detect anti-FFV Gag and anti-FFV Bet antibodies as previously described [55, 74]. Glutathione casein was used to coat 96-well plates (Thermo Fisher Scientific, Waltham, MA) overnight at 4 °C then plates were blocked with casein blocking buffer (0.2% (w/v) casein in PBS and Tween20, Thermo Fisher Scientific). Plates were incubated with BL21 *E. coli*-produced lysates containing GST-tag, GST-Gag-tag, or GST-Bet-tag recombinant proteins (0.25 µg/µl in casein blocking buffer). Cat sera were pre-adsorbed with GST-tag lysate (2 µg/µl) in a 1:50 dilution and then incubated in duplicate (Fig. 7a, b) or triplicate (Additional file 6) with each GST conjugate. The plates were incubated with anti-cat IgG Protein A peroxidase (1:50,000 dilution, Sigma Aldrich). For the substrate reaction, plates were incubated with TMB substrate before stopping the reaction with sulfuric acid. Absorption (optical density, OD) at 450 nm was measured and the mean reactivity for each was used. Detection cutoff values were determined from negative sera as 2 × (mean + 3 standard deviations). A significant number of reactions at the serum dilution used were out of the linear range of the assay. For anti-Gag antibody titrations, sera from days 28, 42, 70, and 168 p.i. were diluted at 1:100, 1:250, 1:500, 1:1000, 1:2500, and 1:5000. Titer was determined as the highest dilution the cat tested positive for anti-Gag antibodies, using the cutoff formula mentioned above.

### FIV Vif antibody capture ELISA

Sera were subjected to an FIV Vif antibody capture ELISA to detect corresponding antibodies in chimeric FFV-Vif-inoculated cats. 96-well plates were coated with 2 ng/µl Vif antigen and incubated overnight at 4 °C. Mouse Vif monoclonal antibody (obtained from Dr. Chris Grant, Custom Monoclonals International, Sacramento, CA) was used as a positive control. After blocking, cat sera (1:100 dilution) or Vif monoclonal antibody (10 ng/µL) were applied in duplicates, then goat anti-cat (or anti-mouse) IgG-HRP (MP Biomedicals, Santa Ana, CA) was used as secondary antibody (1:1000 dilution). TMB reagent was used for the substrate reaction then stopped with sulfuric acid before measuring absorption (450 nm). For detection cutoff, the mean negative sera absorbance readout was used in the following formula: mean + (2 × standard deviation). A number of reactions at the serum dilution used were out of the linear range of the assay.

## Additional files

**Additional file 1.** Rescue of Vif-deficient FIV and Bet-deficient FFV by FIV Vif and FFV Bet. **A** Vif-deficient FIV plasmid DNA was co-transfected with plasmids expressing FIV Vif or FFV Bet together with different feA3 restriction factors as given in the legend (left panel). Empty vector pcDNA3.1 served as control. Two days after transfection, cell-free supernatants were used to infect FIV reporter cells and luc activity induced by FIV infection was measured two days p.i. Titers are expressed as luc values of a representative experiment. **B** The Bet-deficient FFV genome pCF7-BBtr was co-transfected with plasmids expressing untagged and V5-tagged FFV Bet or two different amounts of FIV Vif expression plasmid together with the major FFV-restricting feA3Z2b-HA as shown below the bar diagram (right panel). Empty vector pcDNA3.1 served as control. Two days after transfection, cell-free supernatants were titrated in triplicate using FFV reporter cells as described in the “Methods” section and are expressed as focus-forming units (FFU) per ml inoculum of a representative experiment. Error bars represent the standard deviation.

**Additional file 2.** Partial genome sequences from pCF7-Vif-4 and the stop mutations of the in vitro-selected FFV-Vif variants. The Trp codon and the downstream G residue (TGGG) ~ 130 bp upstream of the *vif* coding sequence are in bold face letters and underlined. In pCF7-Vif W/*1 (in blue), the mutation is from TGG to TGA and for mutant W/*2 (in green) the mutation is from TGGG to TAGA, with both mutations resulting in a Trp (W) to Stop (*) mutation (W/*) as indicated. The *bet* nucleotide sequence is in black, the linker sequence in pink with recognition sites for *Nhe*I (in brown) and *Sac*II (in light violet). The *vif* gene is marked in blue with the authentic Met start codon in bold. The Bet^tr^Vif fusion protein is highlighted in yellow with the amino acids color-coded as described above for the genes. The Met residue 14 amino acids upstream of the authentic *vif* start codon is highlighted in bold and underlining. The C-terminal amino acid sequence of *tas* is highlighted in red.

**Additional file 3.** Mutations in *Tas* generated during the analysis of the upstream ATG do not affect Tas-mediated LTR transactivation. The LTR promoter-based luc reporter construct pFeFV-LTR-luc [73] was cotransfected into HEK 293T cells together with a CMV-IE-driven FFV Tas expression construct, the empty control pcDNA3.1 and proviral genomes pCF-7, pCF7-Vif-4, pCF7-Vif W/*1, and pCF7-Vif W/*2, and their engineered M/T and M^+^ variants. Two days post transfection, luc activity induced by FFV Tas expression was measured in duplicates. Data from a representative experiment normalized to co-expressed β-gal are expressed in a logarithmic bar diagram.

**Additional file 4.** Titers of pCF-7, pCF7-Vif-4 and engineered pCF7-Vif W/*1 and pCF7-Vif W/*2 variants. Plasmid pCF-7, pCF7-Vif-4, pCF7-Vif W/*1, and pCF7-Vif W/*2 and their engineered M/T and M^+^ variants were transfected into HEK 293T cells and 2 days post-transfection, cell-free supernatants were inoculated on CrFK cells and serially passaged every **A** 60 and **B** 84 h p.i. FFV titers were determined in duplicate using FeFAB reporter cells and are shown as bar diagrams for the different passages. Error bars represent the standard deviation.

**Additional file 5.** Date FFV was first detected by PCR and ELISA in experimentally infected cats. Day of first detection of FFV genomic DNA by qPCR with indeterminate and clear positive results (two left columns) and nested PCR (nPCR, middle column) after experimental infection with either wild-type FFV (WT), FFV-Vif W/*1 chimera (CH), chimera then wild-type FFV (CH1WT and CH2WT), twice with FFV-Vif W/*1 chimera (CH3CH and CH4CH), or sham inoculation in naïve cats. In addition, first detection of FFV Gag and Bet, and FIV Vif antibodies by ELISA is displayed correspondingly (right columns). Hyphens (-) mark negative results due to absence of reactivity.

**Additional file 6.** All cats infected with wild-type FFV and FFV-Vif W/*1 developed FFV Gag-specific immunoreactivity. A GST-capture ELISA was performed to evaluate antibody response to FFV infection. Anti-Gag reactivity (1:50 dilution) at the final time point for each animal is shown. All animals exposed to wild-type FFV (red bars) or FFV-Vif W/*1 (blue bars) seroconverted against Gag antigen and for many of these samples, reactivity is out of the linear range. Naïve animals (black bars) remained below the cutoff for detection (black dotted line). Black and blue striped bars denote chimeric animals re-inoculated with wild-type virus (CHxWT). Error bars represent standard deviation. POS = positive control, NEG = negative control, H2O = absolute negative (water) control.

## Abbreviations

FV: foamy virus(es)
FFV: feline foamy virus
LV: lentivirus(es)
FIV: feline immunodeficiency virus
HIV: human immunodeficiency virus
A3: APOBEC3 proteins
feA3: feline A3 proteins
WT: wild-type pCF-7 inoculated group
CH: chimeric pCF7-Vif W/*1 inoculated group
N: naïve control group
PBMC: peripheral blood mononuclear cell(s)
W/*: Trp/stop mutation
p.i: post-infection
i.m: intramuscular(ly)
i.v: intravenous(ly)
Py: pyrimidine residue
CSU: Colorado State University
MOI: multiplicity of infection
luc: luciferase
PR: protease
CrFK: Crandell feline kidney cell(s)
HEK: human embryonic kidney cell(s)
FFU: focus-forming units
Thr: threonine
Trp: tryptophan
CPE: cytopathic effect
w/v: weight per volume
SPF: specific-pathogen free
