# Supplementary figures and images for: Replacement of feline foamy virus bet by feline immunodeficiency virus vif yields replicative virus with novel vaccine candidate potential
Source: Retrovirology. 2018 May 16;15:38. doi: 10.1186/s12977-018-0419-0 (PMC5956581; doi:10.1186/s12977-018-0419-0)

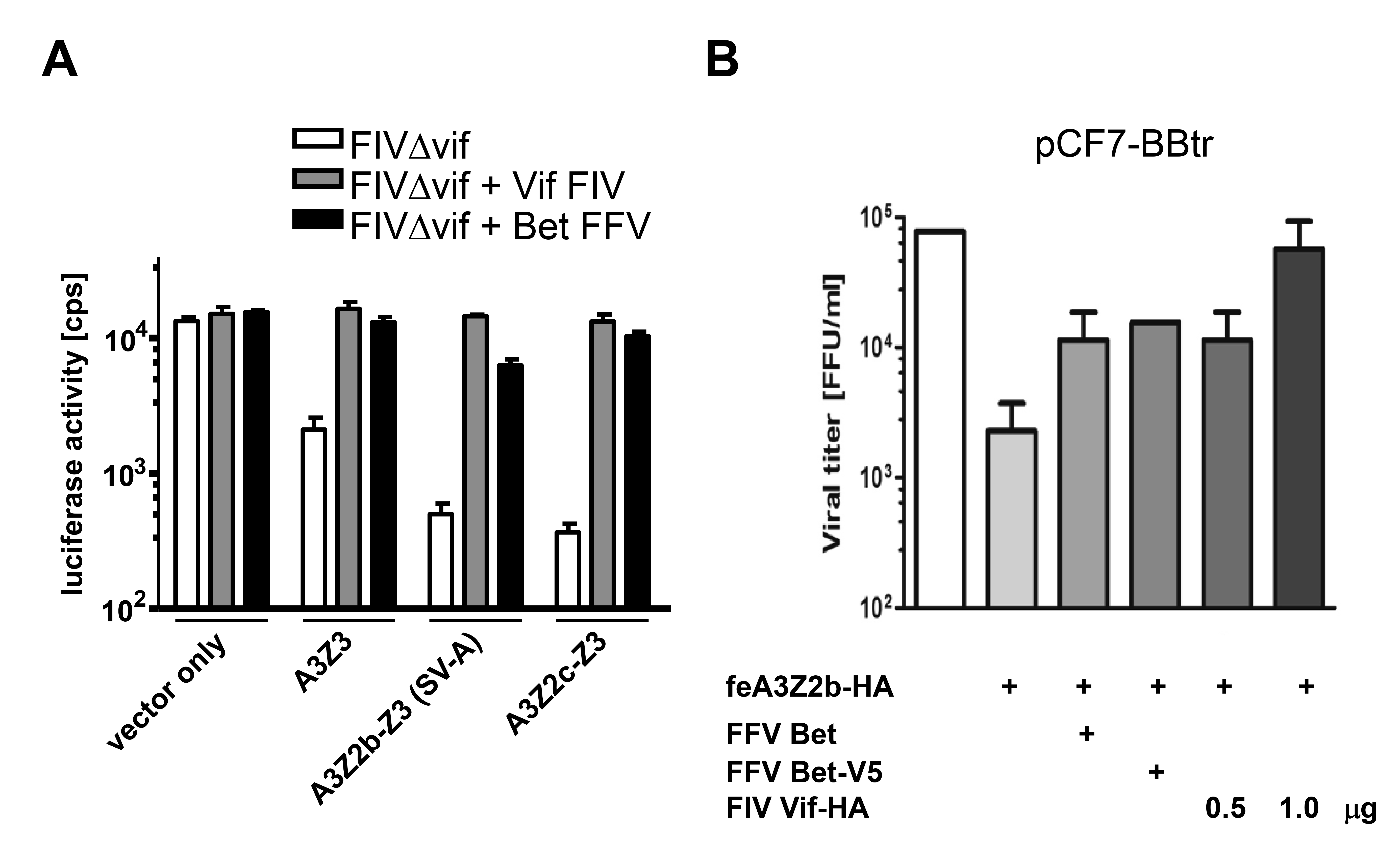

Supplement: Supplementary file 1 — Additional file 1. Rescue of Vif-deficient FIV and Bet-deficient FFV by FIV Vif and FFV Bet. A Vif-deficient FIV plasmid DNA was co-transfected with plasmids expressing FIV Vif or FFV Bet together with different feA3 restriction factors as given in the legend (left panel). Empty vector pcDNA3.1 served as control. Two days after transfection, cell-free supernatants were used to infect FIV reporter cells and luc activity induced by FIV infection was measured two days p.i. Titers are expressed as luc values of a representative experiment. B The Bet-deficient FFV genome pCF7-BBtr was co-transfected with plasmids expressing untagged and V5-tagged FFV Bet or two different amounts of FIV Vif expression plasmid together with the major FFV-restricting feA3Z2b-HA as shown below the bar diagram (right panel). Empty vector pcDNA3.1 served as control. Two days after transfection, cell-free supernatants were titrated in triplicate using FFV reporter cells as described in the “Methods” section and are expressed as focus-forming units (FFU) per ml inoculum of a representative experiment. Error bars represent the standard deviation. [file 12977_2018_419_MOESM1_ESM.tif]

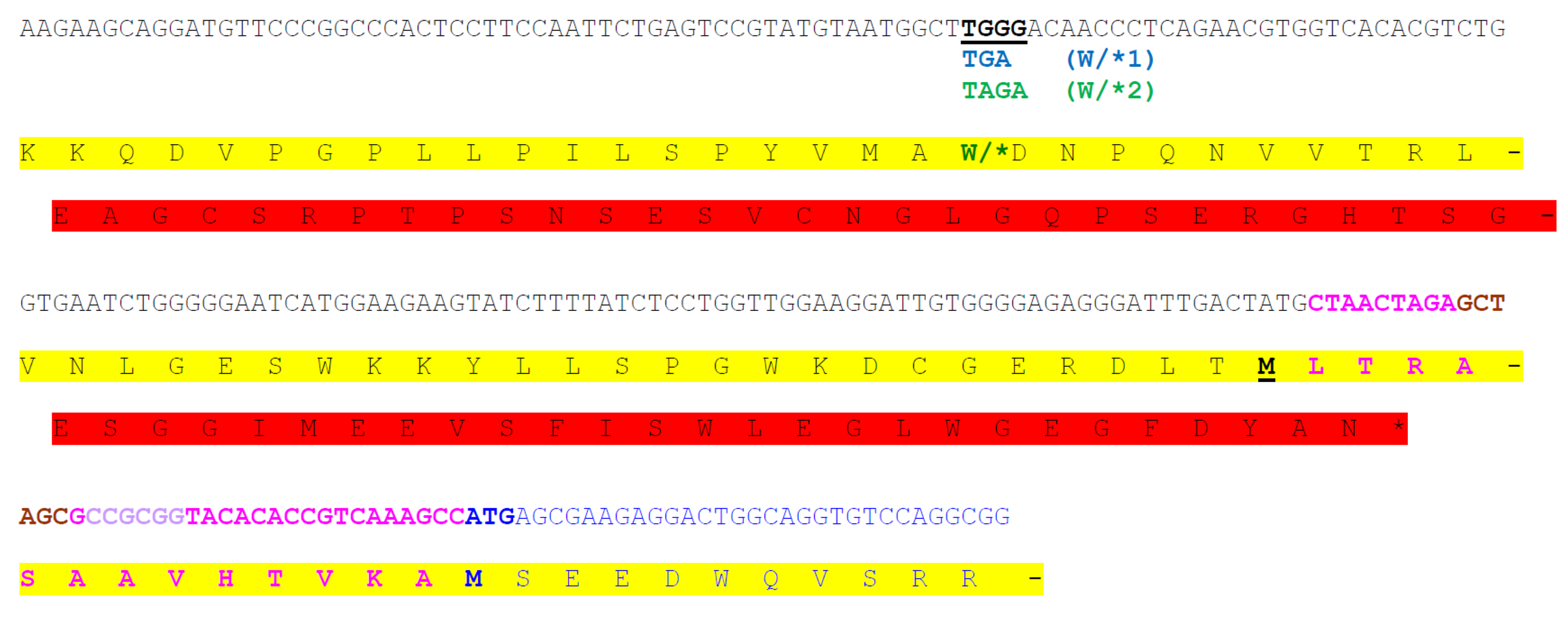

Supplement: Supplementary file 2 — Additional file 2. Partial genome sequences from pCF7-Vif-4 and the stop mutations of the in vitro-selected FFV-Vif variants. The Trp codon and the downstream G residue (TGGG) ~ 130 bp upstream of the vif coding sequence are in bold face letters and underlined. In pCF7-Vif W/*1 (in blue), the mutation is from TGG to TGA and for mutant W/*2 (in green) the mutation is from TGGG to TAGA, with both mutations resulting in a Trp (W) to Stop (*) mutation (W/*) as indicated. The bet nucleotide sequence is in black, the linker sequence in pink with recognition sites for NheI (in brown) and SacII (in light violet). The vif gene is marked in blue with the authentic Met start codon in bold. The BettrVif fusion protein is highlighted in yellow with the amino acids color-coded as described above for the genes. The Met residue 14 amino acids upstream of the authentic vif start codon is highlighted in bold and underlining. The C-terminal amino acid sequence of tas is highlighted in red. [file 12977_2018_419_MOESM2_ESM.tif]

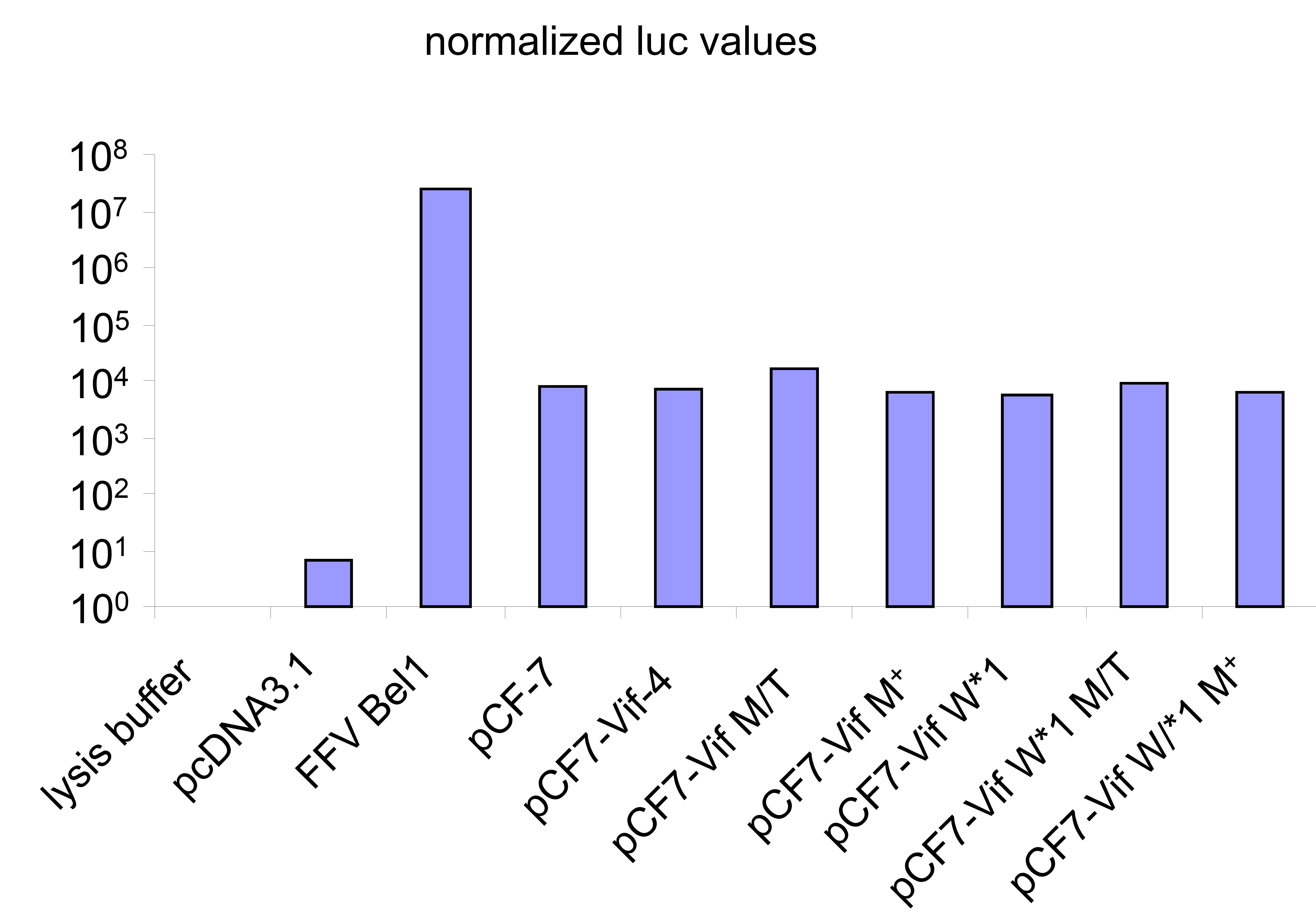

Supplement: Supplementary file 3 — Additional file 3. Mutations in Tas generated during the analysis of the upstream ATG do not affect Tas-mediated LTR transactivation. The LTR promoter-based luc reporter construct pFeFV-LTR-luc [73] was cotransfected into HEK 293T cells together with a CMV-IE-driven FFV Tas expression construct, the empty control pcDNA3.1 and proviral genomes pCF-7, pCF7-Vif-4, pCF7-Vif W/*1, and pCF7-Vif W/*2, and their engineered M/T and M+ variants. Two days post transfection, luc activity induced by FFV Tas expression was measured in duplicates. Data from a representative experiment normalized to co-expressed β-gal are expressed in a logarithmic bar diagram. [file 12977_2018_419_MOESM3_ESM.tif]

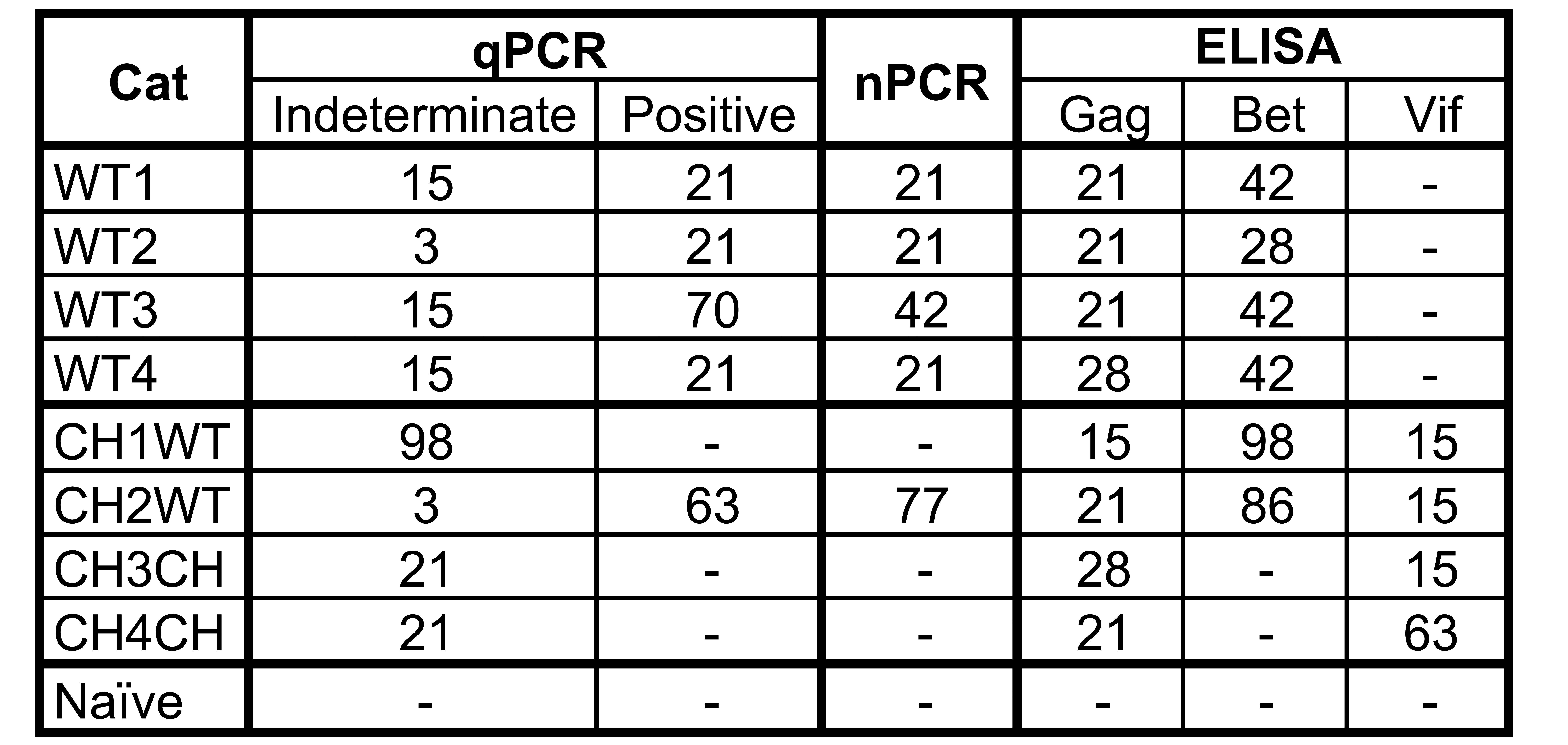

Supplement: Supplementary file 5 — Additional file 5. Date FFV was first detected by PCR and ELISA in experimentally infected cats. Day of first detection of FFV genomic DNA by qPCR with indeterminate and clear positive results (two left columns) and nested PCR (nPCR, middle column) after experimental infection with either wild-type FFV (WT), FFV-Vif W/*1 chimera (CH), chimera then wild-type FFV (CH1WT and CH2WT), twice with FFV-Vif W/*1 chimera (CH3CH and CH4CH), or sham inoculation in naïve cats. In addition, first detection of FFV Gag and Bet, and FIV Vif antibodies by ELISA is displayed correspondingly (right columns). Hyphens (-) mark negative results due to absence of reactivity. [file 12977_2018_419_MOESM5_ESM.tif]

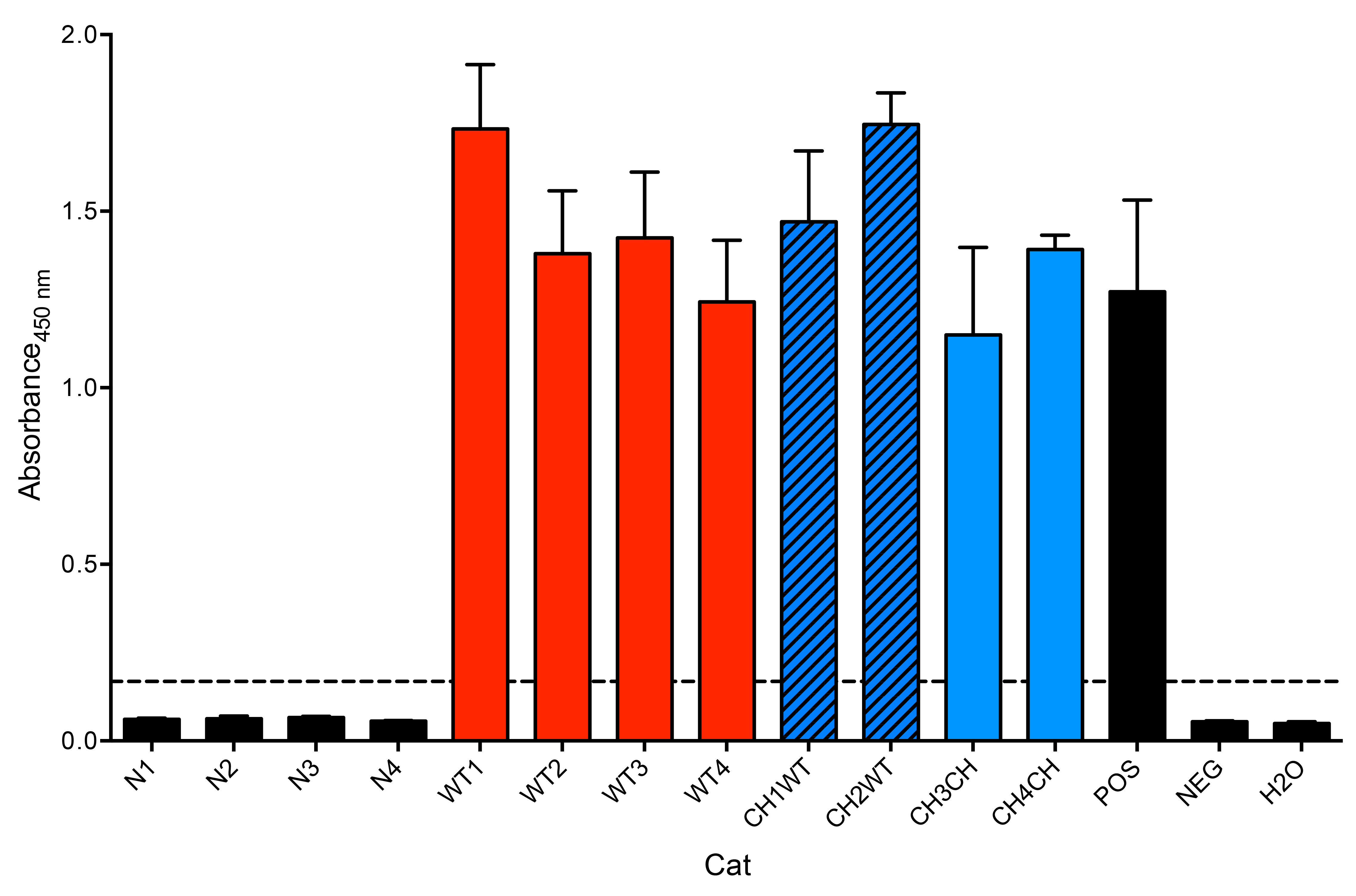

Supplement: Supplementary file 6 — Additional file 6. All cats infected with wild-type FFV and FFV-Vif W/*1 developed FFV Gag-specific immunoreactivity. A GST-capture ELISA was performed to evaluate antibody response to FFV infection. Anti-Gag reactivity (1:50 dilution) at the final time point for each animal is shown. All animals exposed to wild-type FFV (red bars) or FFV-Vif W/*1 (blue bars) seroconverted against Gag antigen and for many of these samples, reactivity is out of the linear range. Naïve animals (black bars) remained below the cutoff for detection (black dotted line). Black and blue striped bars denote chimeric animals re-inoculated with wild-type virus (CHxWT). Error bars represent standard deviation. POS = positive control, NEG = negative control, H2O = absolute negative (water) control. [file 12977_2018_419_MOESM6_ESM.tif]
